# Synthesis, Catalytic Properties and Application in Biosensorics of Nanozymes and Electronanocatalysts: A Review

**DOI:** 10.3390/s20164509

**Published:** 2020-08-12

**Authors:** Nataliya Stasyuk, Oleh Smutok, Olha Demkiv, Tetiana Prokopiv, Galina Gayda, Marina Nisnevitch, Mykhailo Gonchar

**Affiliations:** 1 Institute of Cell Biology, National Academy of Sciences of Ukraine, 79005 Lviv, Ukraine; stasukne@nas.gov.ua (N.S.); smutok@cellbiol.lviv.ua (O.S.); demkivo@nas.gov.ua (O.D.); t.prokopiv@nas.gov.ua (T.P.); galina.gayda@nas.gov.ua (G.G.); 2 Department of Biology and Chemistry, Drohobych Ivan Franko State Pedagogical University, 82100 Drohobych, Ukraine; 3 Faculty of Veterinary Hygiene, Ecology and Law, Stepan Gzhytskyi National University of Veterinary Medicine and Biotechnologies, 79000 Lviv, Ukraine; 4 Department of Chemical Engineering, Ariel University, Kyriat-ha-Mada, Ariel 4070000, Israel; marinan@ariel.ac.il

**Keywords:** nanoparticle, nanocomposite, nanozyme, synthesis, catalytic properties, nano-peroxidase, nanooxidase, nanolaccase, electronanocatalyst, amperometric (bio)sensors

## Abstract

The current review is devoted to nanozymes, i.e., nanostructured artificial enzymes which mimic the catalytic properties of natural enzymes. Use of the term “nanozyme” in the literature as indicating an enzyme is not always justified. For example, it is used inappropriately for nanomaterials bound with electrodes that possess catalytic activity only when applying an electric potential. If the enzyme-like activity of such a material is not proven in solution (without applying the potential), such a catalyst should be named an “electronanocatalyst”, not a nanozyme. This paper presents a review of the classification of the nanozymes, their advantages vs. natural enzymes, and potential practical applications. Special attention is paid to nanozyme synthesis methods (hydrothermal and solvothermal, chemical reduction, sol-gel method, co-precipitation, polymerization/polycondensation, electrochemical deposition). The catalytic performance of nanozymes is characterized, a critical point of view on catalytic parameters of nanozymes described in scientific papers is presented and typical mistakes are analyzed. The central part of the review relates to characterization of nanozymes which mimic natural enzymes with analytical importance (“nanoperoxidase”, “nanooxidases”, “nanolaccase”) and their use in the construction of electro-chemical (bio)sensors (“nanosensors”).

## 1. Introduction: Definition of Nanozymes, Classification, Advantages vs. Natural Enzymes, and Potential Practical Applications

Enzymes are biological catalysts that play a key role in biological processes. They have long been an indispensable tool in many chemical and biotechnological processes that are widely used in food processing, industry, agriculture and medicine. Natural enzymes (except for ribozymes) are proteins and are responsible for almost all biochemical reactions in living organisms. They are characterized by high selectivity and extremely high catalytic activity (Table 1). However, natural enzymes tend to have limited chemical and biological stability as well as a high cost due to complicated technologies employed for their isolation and purification from biological sources. Although modern molecular technologies (gene cloning, genetic and protein engineering, etc.) significantly facilitate these procedures, obtaining highly purified enzymes in commercial quantities is still a key challenge for practical enzymology.

Artificial substitutes for enzymes were invented toward the end of the twentieth century [1,2]. Artificial enzymes include cyclodextrins with catalytic activity, abzymes (antibodies with catalytic activity), synzymes (synthetic enzymes) and aptamers (DNAzymes and RNAzymes). They are usually more stable than natural enzymes but are inferior in their catalytic activity and preparation costs due to complicated synthesis technologies. As a rule, artificial enzymes are unfortunately not as substrate specific as natural enzymes.

Nanozymes (NZs) are the newest class of functional nanomaterials [3,4,5,6] that have enzyme-like activity. They possess increased stability and greater availability due to their simpler preparation technologies. Described nanoscale materials include catalysts with different reaction specificities. They are mainly oxidoreductases: peroxidase [7], haloperoxidase, catalase, glucose oxidase, sulfite oxidase, superoxide dismutase (SOD), laccase, monoxygenase, CO oxidase, ferritin ferrooxidase [8], different hydrolases (phosphatase, phosphotriesterase, chymotrypsin, carbonic anhydrase), as well as proteases, endonucleases, DNA-ases, NO synthases, etc. [9,10,11]. 

The term “nanozyme” was defined by Wei and Wang in 2013 [3], although the first exciting discovery of Fe_3_O_4_-based ferromagnetic nanoparticles (NPs) with peroxidase(PO)-like catalytic activity was made in 2007 by Gao [12], as cited by Huang [6]. In our opinion, Prussian Blue is another nanomaterial candidate for which catalytic activity toward hydrogen peroxide was proven for the first time (see, for example, a review by Karyakin in 2001 [13]). 

The most important advantage of nanozymes is their size/composition-dependent activity. This allows the design of materials with a broad range of catalytic activity simply by varying shape, structure, and composition. NZs also have unique properties compared to other artificial enzymes, including large surface areas which significantly facilitate their further modification and bioconjugation. The ability of nanomaterials to self-assemble is also a very important characteristic for biology and medicine, due to easier incorporation of biological components into the nanomaterial’s structure (Table 2).

There is no official classification of NZs. Huang et al. (2019) [6] proposed dividing NZs into two categories: (1) oxidoreductases (oxidases, peroxidase, catalase, superoxide dismutase, and nitrate reductase); (2) hydrolases (nucleases, esterases, phosphatases, proteases, and silicatein). Such a classification could easily be expanded upon the discovery of novel NZs with other catalytic activities, similarly to other natural enzymes allocated to Enzyme Committee (EC) classes.

Additional problems arise when attempting to classify NZs according to the chemical structure of the catalytic nanocomposite (see Table 1 from [6]). Such classification will be rather cumbersome, due to the broad chemical diversity of NZs. For example, the list of known NZs includes: NPs of noble and transient metals, their hybrid forms, carbon NPs (graphene, carbon nanotubes, fullerene), metal oxides, metal sulfides and tellurides, carbon nitride, quantum dots, 2D-nanomaterials with confined single metal and nonmetal atoms [14], Prussian Blue NPs, polypirrole, hemin micelles and many nanomaterials that have been functionalized or modified by organic ligands [3,5].

**Figure 1:**
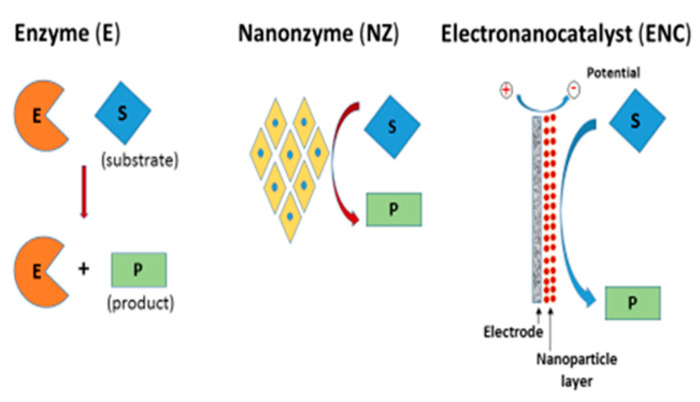
Principal scheme of catalytic action of enzyme, nanozyme and electronanocatalyst.

In many cases, the term “NZ” or “enzyme-mimicking activity” is used without a sufficiently strong reason. On our opinion, these terms can be used properly only for materials whose enzyme-mimicking activity has been proven in solution. In most papers, the catalytic activity was revealed only after applying a potential to an electrode modified by the tested nanocomposite. In this case, the catalytic nanomaterial should be named an “electrocatalyst” (“electronanocatalyst”), but not, strictly speaking, a “nanozyme” or “enzyme mimic” (Figure 1). We therefore propose naming a catalytic nanomaterial as a “NZ” (nanooxidase, nanoperoxidase, nanocatalase, nanolaccase, and so on) only when the nanomaterial exhibits the enzyme-like catalytic activity in solution/suspension, without applying an electrochemical potential. 

Although in most cases the catalytic efficiency of NZs is lower compared to natural analogues, it was reported that some NZs can compete with the enzymes. For example, the highest values for any NZ reported to date belong to MnFe_2_O_4_ with a nanooctahedron morphology, whose turnover number (k_cat_) and catalytic efficiency (k_cat_/K_M_) in the oxidation of 3,3´,5,5´-tetramethylbenzidine (TMB) are 8.34 × 10^4^ s^−1^ and 2.21 × 10^9^ M^−1^·s^−1^, respectively [15,16]. 

It is worth noting that in many (or even most) publications, the catalytic activity is presented only by a V_max_ value, without indicating the catalyst concentration. This is problematic, since the V_max_ depends on the catalyst (enzyme) concentration (V_max_ = [E]·k_cat_). In order to compare the intrinsic kinetic parameters of different nanocatalysts with each other or with natural enzymes, a catalytic constant (a turnover number, k_cat_) must be used, or at least the V_max_ value, but in conjunction with the concentration at which the maximal reaction rate of the nanocatalyst was determined. Valuable recommendations on this problem and standardization of experimental protocols for measurement of enzyme-mimicking activity are presented by Jiang et al. [17]. 

As a consequence of their catalytic activity, NZs can be applied for practical use in scientific research, biotechnology and food industries, agriculture, degradation of environmental pollutants (wastewater treatment, degradation of chemical warfare agents), clinical diagnostics and pharmacology [6,17,18,19]. In addition to their use as diagnostic tools, nanozymes are promising catalytic components of therapeutic drugs [6,8,9,10,11,20,21]. Due to the biocompatibility and magnetic properties of some NZs, they can be used for targeted treatment of malignancies. NZs have the potential for serving as antioxidants in the treatment of autoimmune, Alzheimer’s and Parkinson’s diseases, and for application as antibacterial agents. Using NZs in the construction of biosensors [18,19,22,23,24] and biofuel cells is very promising, where high catalytic activity, chemical and biological stability, nanoscale size of catalytic elements, and more cost-effective preparation are the most important challenges.

In the future, we can expect a sophisticated design of NZs in silico which will allow them to compete with natural enzymes in catalytic efficiency and selectivity. NZs sensitive to regulation by low-molecular effectors (activators and inhibitors) and able to catalyze cascade reactions will be created, and their catalytic behavior will be adapted to different environmental conditions. Large scale production will also be developed, based on cost-effective physicochemical methods and “green synthesis” approaches.

To date, over 300 types of nanomaterials were found to possess intrinsic enzyme-like activity. A large variety of NZs (or ENCs) simultaneously exhibit dual- or multienzyme mimetic activity [10]. Several reviews that summarize the new data concerning synthesis, characterization, bioanalytical and medical application of NZs are published every year, and the number of these reports is rising in a geometrical progression [10,23,24]. Thus, “nanozymology”, as a new field of science connecting nanotechnology and enzymology, has great potential for further development and for many practical future applications [10,17].

The current paper is devoted to reviewing the recent advantages and current challenges of using NZs (or electronanocatalysts) as catalytic and recognition elements in biosensors. The catalytic performance of NZs is characterized, a critical point of view on catalytic parameters of NZs described in scientific papers is presented, and typical mistakes are analyzed. The central part of the review relates to characterization of NZs which mimic natural enzymes with analytical importance (“nanoperoxidase”, “nanooxidases”, “nanolaccase”) and their use in the construction of electro-chemical (bio)sensors (“nanosensors”).

## 2. Methods for the Synthesis of Catalytic Nanomaterials

Catalytic nanomaterials are known to possess unique properties compared with natural enzymes [25]. It was shown that activities of NZs are greatly dependent on the chemical structure, particle size, shape and surface morphology, which could be affected by charges, coatings, dopings, loadings and external fields. The morphology of synthesized NZs can be controlled due to rapid development of nanotechnology techniques [16,26].

**Figure 2:**
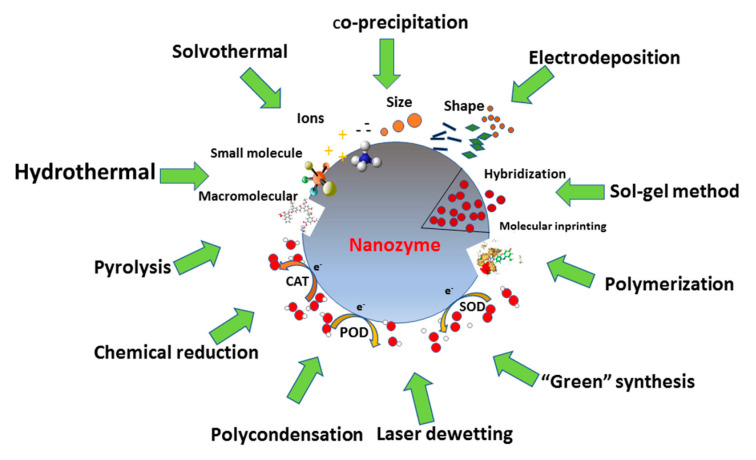
Principal scheme of the methods for nanocatalyst synthesis.

This section summarizes different methods for preparation of nanomaterials possessing catalytic, mainly electrocatalytic, properties, in particular hydrothermal and solvothermal methods, co-precipitation and sol-gel methods, etc. Their applications for construction of biosensors are also described. Figure 2 presents the principal scheme illustrating methods of nanocatalyst synthesis. The kinetic parameters for different types of nanocatalysts are summarized in Tables 3–11. Table 3 relates to the effect of synthetic methods on the structural and functional properties of ENCs.

### 2.1. Hydrothermal and Solvothermal Methods

The main characteristics of the obtained NZs can be changed in accordance with the method chosen for NZ synthesis. The most promising techniques for synthesizing nanomaterials are the hydrothermal and solvothermal methods. Low-cost nanocrystals with well-controlled dimensions can be obtained using the proposed approaches [26].

A series of spinel-type Co_x_Ni_1_-xFe_2_O_4_ (x = 0, 0.2, 0.4, 0.5, 0.6, 0.8, 1.0) nanocatalysts were synthesized using the solvothermal method, where ethylene glycol served as the solvent. The obtained NZs were employed as enzyme mimics for the detection of H_2_O_2_. The Co_0.5_Ni_0.5_Fe_2_O_4_/CPE exhibited a wider linear range and a higher sensitivity compared with H_2_O_2_-selective enzymatic biosensors based on spinel-type ferrites, indicating a promising future for Co_x_Ni_1_-xFe_2_O_4_ as enzyme mimics for the construction of chemosensors [27]. The surface morphology of the synthesized Co_x_Ni_1_-xFe_2_O_4_ ENCs was studied and a diameter of 70 to 130 nm was observed. The small size of the nanocatalysts greatly increases the effective surface area for electrocatalytically active sites, and thus improves the physicochemical properties of the amperometric biosensor [27] (Table 3).

Solid spherical crystals of CuO (CuO-ENCs) having an average size of 45 nm were prepared by thermal decomposition of a precursor-Cu-based metal-organic gel (Cu-MOG). It was shown that the obtained CuO-ENCs possessed high electrocatalytic activity in glucose oxidation and exhibited PO-like catalytic ability. The CuO-ENC can also be used as a biomimetic for detection of cholesterol [28]. CuO-ENCs have high Brunauer-Emmett-Teller (BET) surface area of 20.16 m^2^·g^−1^, high pore volume of 0.11 cm^3^·g^−1^ and the average pore diameter of ~2.5 nm [29]. In another example, two kinds of carbon-based nanocatalysts with a size of 100–150 nm were synthesized using a combination of two methods, a thermal method and a solid-state reaction, from the zeolitic imidazolate framework-8 (ZIF-8) [30,31]. The carbon cubic nanomaterial (labelled HCC) with the hollow structure was obtained by chemically etching ZIF-8 with tannic acid, followed by a calcination process. The carbon cubic nanomaterial with the porous surface (labelled PCC) was obtained by direct pyrolysis. The two types of synthesized NZs, HCC and PCC, possess a BET specific surface area of 356 and 756 m^2^·g^−1^, respectively [30].

Electrochemical detection of glucose and fructose based on gold nanoparticles (AuNPs) deposited onto graphene paper has recently been proposed. These nanostructures were synthesized via two approaches: thermal and laser dewetting processes [32]. Gold nanostructures obtained by both methods exhibited major differences in their particle morphology. Both types of AuNPs were examined by their ability to oxidize glucose and fructose. The corresponding analytical characteristics of the constructed chemosensors are presented in Table 3.

It was shown that the BET of PCC was higher than that of HCC, indicating that PCC had more specific area for analyte adhesion (Table 3) [30].

The gold/cobalt (Au/Co) bimetallic nanocomposite-decorated hollow nanoporous carbon framework (Au/Co@HNCF) was synthesized as an ENC by thermal pyrolysis at 900 °C of the Au (III)-etching zeolitic imidazolate framework-67 (ZIF-67) [33]. An ultrasensitive electrochemical biosensor was developed to identify low levels of uric acid in human serum. Scanning electron microscopy images showed that the Au (III)-etching ZIF-67 has a dodecahedron shape (Table 3). The Au/Co@HNCF biosensor (7.88 cm^2^) exhibited a significantly higher electrochemical active surface area than the bare glassy carbon electrode (GCE, 0.0155 cm^2^), indicating the existence of abundant active sites on the Au/Co@HNCF modified layers.

### 2.2. Chemical Reduction

Chemical reduction is the most frequently used method, due to its rapidity and simplicity. This technique enables producing NPs in which the morphology and particle size distribution are controlled by changing the molar concentration of the reactants, the reductant type and the reaction temperature [34]. The critical factor in achieving high chemical reduction is choosing the appropriate reductants. Reduction of metal salts requires reactivity of the reduction agent to the redox potential of the metal. The obtained particles are small if the reaction rate during the synthesis process is too fast [35]. However, if the reaction rate is too slow, particle aggregation may occur [36]. The synthesis of hollow copper sulfide nanocubes (h-CuS NCs) was conducted via the chemical reduction method [37]. The average particle size was 50 nm and the specific surface area was 57.84 m^2^·g^−1^, which is larger than that of general solid CuS (34.76 m^2^·g^−1^). h-CuS NCs exhibited promising signs for high enzyme-mimicking catalytic activity (Table 3).

The chemical reduction method has been used for the synthesis of a peroxidase (PO)-like NZ based AuNPs in combination with *Pseudomonas aeruginosa*–specific aptamer [38]. The average particle size was ~20 ± 3 nm. The proposed bioelectrode structure can be used for detection of other bacterial pathogens in water or biological fluids.

ENC-carbon supported Pt-NiNPs stabilized with oleylamine (Pt-Ni) were synthesized by chemical reduction of metal salts [39]. The synthesized ENCs were near-spherical in shape, with a mean diameter of 8.4 nm. The ENCs exhibited an active electrochemical surface area of 2.3 m^2^·g^−1^, which is lower than that of the etched particles without surfactant (Table 3).

Metal-organic frameworks (MOFs) are composed of metal ions as nodes and organic ligands as linkers and have diversified and tailorable structures. MOFs have high surface area and porosity, exposed active sites and good biocompatibility, and for these reasons they attact wide attention as a blooming alternative material. A multifunctional artificial enzyme was synthesized through a combination of the chemical reduction method and electrodeposition technique by modifying PtNPs on the metalloporphyrin MOF [40]. In the Pt@PMOF(Fe) complex, PMOF(Fe) could prevent the aggregation of PtNPs, leading to high stability of the Pt NPs. Furthermore, PtNPs exhibited catalase-and PO-like activities. In another example, oxidase-like nanosheets were prepared [41] using the chemical reduction method modified by Hummers et al. [42], in which histidine enantiomers served as both a reducing agent and a protecting ligand. Compared to the previously developed strategies, this synthesis method is very rapid and mild and does not require heating, pressuring, and special media. It is a mild synthesis strategy, where only two reactants of HAuCl_4_ and the His enantiomers are involved, without additional catalysts or templates. The oxidase-like and electrocatalytic activity of His@AuNCs/RGO was evaluated for the determination of nitrite (Table 3). The developed method was used for the detection of nitrites in real samples [41].

The green synthesis procedure was recently proposed as an important method for producing inorganic NZs. It is well known that living organisms can produce substances that act as reducing agents [6,11,16]. For example, Han et al. proposed a new environmentally friendly technology for synthesis of MoO_3_ NZs in which green algae (*Enteromorpha prolifera*, EP) were used as the reducing agent [43]. The obtained MoO_3_ NZs revealed excellent PO-like activity and were used, together with GOx, for colorimetric detection of glucose in human serum. In another work, Han and co-authors [44] used bovine serum albumin (BSA) as a biotemplate for Co_3_O_4_ NZs synthesis. The obtained NZs had a spherical morphology with an average diameter of 60 nm and exhibited catalase-like activities.

### 2.3. Sol-gel Method

In this method, a gel-like network containing both a liquid phase and a solid phase is formed. The crystallinity, morphology and magnetic properties of the NZs can be controlled by choosing an appropriate complexing agent, concentration and type of chemical additives, and temperature conditions [28].

The synthesis of PtNPs polyaniline (PAni) hydrogel heterostructures was performed via the sol-gel method [45]. Phytic acid was used as a complexing agent. The PtNPs loaded into the hydrogel matrix were found to act as active catalysts for the oxidation of H_2_O_2_. The obtained PtNP/PAni hydrogel had a 3D hierarchical structure consisting of connected PAni nanofibers with diameters of approximately 100 nm (Table 3). The porous structure of the PAni hydrogel allows immobilization of concentrated enzyme solutions. Since water-soluble molecules can penetrate through the hydrogel, the PtNPs preserve their ability to catalyze glucose oxidation.

### 2.4. Co-Precipitation

Co-precipitation is a fast method for the synthesis of different types of nanocatalysts. Co-precipitation is an excellent choice when higher purity and better stoichiometric control are required [46]. Xuan Cai et al. [47] prepared two kinds of nanomaterials by applying the co-precipitation synthesis method: carbon spheres (Mn-MPSA-HCS) and hollow carbon cubic materials (Mn-MPSA-HCC) with a size of 100 to 200 nm, respectively, for O_2_^•−^ sensing (Table 3). Physicochemical characterization of the obtained nanocatalyst demonstrates that the Mn_x_(PO_4_)_y_ monolayer was homogeneously dispersed on the surface of the carbon structures without visible size or morphologies, thus providing numerous active sites for reaction of analytes. The proposed method can be adapted as a universal strategy for fabricating transition metal phosphates with all kinds of shapes and sizes for different applications and is particularly promising for biosensing. In another example, Dashtestani et al. [48] used a combination of two methods for nanocomposite synthesis: chemical reduction of HAuCl_4_ and co-precipitation of the obtained gold nanoparticles (GNPs) with the copper(II) complex of cysteine (GNP/Cu-Cys). The combination of GNPs and Cu-Cys complex increased the electrochemical signal toward O_2_^•^ (Table 3).

The sensitive electrochemical biosensor based on dual aptamers was proposed for detection of cardiac troponin I (cTnI). The biosensor included DNA nanotetrahedron (NTH) capture probes and multifunctional hybrid nanoprobes. First, the NTH-based Tro4 aptamer probes were immobilized on a screen-printed gold electrode (SPGE) surface through the Au–S bond. Then, the hybrid nanoprobes were prepared using magnetic Fe_3_O_4_ NPs as nanocarriers for immobilization of a cTnI-specific Tro6 aptamer, horseradish peroxidase (PO), PO-mimicking Au@Pt nanozymes and G-quadruplex/hemin DNAzyme [49]. The constructed sensor exhibited a wide linear concentration range (10 pg·mL^−1^ to 100 ng·mL^−1^) and a low LOD (7.5 pg·mL^−1^) for cTnI (Table 3).

### 2.5. Polymerization and Polycondensation

NZs can be obtained either by using insoluble polymers or by cross-linking of a soluble polymer [50]. Mesoporous SiO_2_–L-lysine hybrid nanodisks were synthesized by Han et al. [51] via hydrolyte polycondensation of tetraethylorthosilicate in the presence of CTMB and L-lysine. The prepared hybrid nanodisks have a high surface area (570 m^2^·g^−1^) and ordered mesopores with a size of about 2.9 nm. The obtained hybrid nanodisks possessed excellent biocompatibility to L-lysine. An electrochemical biosensor for superoxide anions (O_2_^•−^) was constructed based on this result.

In another example, Santhosh et al. [52] synthesized composite core-shell nanofibers consisting of gold NPs on poly(methylmethacrylate) (PMMA) by the combination of an electrospinning technique and in situ polymerization of aniline. The average diameter of the PMMA fibers was 400–500 nm. The surface of the fibers was fairly smooth and randomly oriented. The proposed core-shell fibers were used for electrochemical detection of the superoxide anion (O_2_^•−^).

### 2.6. Electrochemical Deposition

Electrochemical deposition is a low-cost method for obtaining metal nanocatalysts. However, it is usually used less often than chemical reduction methods [53]. The process is simple and includes an immersion of a conductive surface into a solution containing ions of the material to be deposited and application of a voltage across the solid/electrolyte interface. In the course of this procedure a charge transfer reaction causes the film deposition [54]. The disadvantage of this method is in impossibility of controlling the morphology. However, it has certain advantages: (1) short synthesis time; (2) absence of chemical reductants or oxidants; (3) absence of undesired byproducts [55]. Electrodeposition is applied using different electrochemical techniques: cyclic voltammetry, potential step deposition method and double-pulse deposition [56]. The particle size can be controlled by adjusting the current density or applied potential and the electrolysis time [57]. Electrodeposition is used for synthesis of nanostructural materials with and without templates. Templates utilized for electrodeposition include porous membranes with a 1D-channel, liquid crystal materials and surfactants [58].

Gallay et al. [59] prepared a hybrid nanocomposite using the electrochemical electrodeposition of RuNPs on the surface of avidin-functionalized multiwalled carbon nanotubes by applying a potential of −0.600 V for 15 s in a 50 ppm ruthenium solution under stirring. The nanohybrid-electrochemical interfaces had excellent PO-like properties toward H_2_O_2_ (Table 3).

A simple method for the electrochemical detection of methylamine and ethanol in real samples of food and alcoholic beverages using PtRu NZ as artificial PO was reported recently [60]. PtRuNPs were synthesized on the surface of a graphite electrode (nPtRu/GE) by electrodeposition of 1 mM RuCl_3_ and a H_2_PtCl_6_-containing solution using the method of cyclic voltammetry in the range of −1000 to +1000 mV with a scan rate of 50 mV·min^−1^ during 10 cycles. The resultant nPtRu/GE turned dark brown-golden due to the formation of nPtRu. Morphological properties of the PtRu-film were studied. It was shown that the average thickness of the deposited layer was 60 nm and it was proven that the obtained layer was nanosized.

Electrosynthesis of AgNP/NCF/GCE was performed by a combination of thermal reduction and electrodeposition methods through thermal synthesis of an electroconductive nitrogen-doped cotton carbon fiber composite (NCF) followed by electrodeposition of AgNPs onto NCF [61]. The developed AgNP/NCF/GCE electrode exhibited outstanding performance toward O_2_^•−^, with a wide linear range and a super-low detection limit.

As mentioned, the applied nanocatalyst synthesis methods can influence their structural and physical properties [25,62,63,64]. The effect of different synthesis methods on structural properties of ENCs with electrocatalytic interfaces are presented in Table 3.

## 3. Catalytic Performance of Nanozymes

Development of new nanocatalysts, including NZs, with higher catalytic activity expands their applications in bioanalytics. As a rule, the activity of NZs is lower than that of enzymes and their catalytic performance strongly depends on: (1) the method of synthesis (pH, reaction time and temperature) [25]; the composition of the nanomaterial [16,65]; the shape, size, dispersity and final morphology [66]; the mass ratio of the components in the nanocomposite [67]; and surface functionalization [40,68,69,70].

The catalytic performance of NZs, as well as their efficiency, is quantitatively estimated by kinetic properties: K_M_, V_max_ (at a defined nanocatalyst concentration), k_cat_, k_cat_/K_M_ ratio, IC_50_ and morphological characteristics: specific BET surface areas, pore volume, pore diameter, crystallite size.

The enzyme mimics follow Michaelis–Menten kinetics, which is similar to natural enzymes. It has been found that the activity of NZs is strongly dependent on the pH, temperature and substrate concentration. The rate of the reaction is generally determined by the reaction extent as a function of time. It is known that K_M_ and k_cat_ are two key parameters for quantifying the catalytic ability of an enzyme, where K_M_ characterizes the affinity of the enzyme to its substrate. The main manifestation is that low K_M_ values reflect high affinity of the enzyme for the substrate. V_max_ describes the reactivity of the enzyme (at a fixed concentration) when saturated with the substrate. K_M_ and k_cat_ are therefore among the important reference standards for judging the superiority of an enzyme vs. a NZ [27]. Different substrates (chromogenic, fluorogenic or chemiluminescent) are used for experimental detection of the enzyme-like activity of NZs. It was shown that His@AuNCs/reduced graphene oxide (His@AuNCs/RGO) exhibits phenol oxidase mimic activity and possesses a low K_M_ value (0.031 mM) with a V_max_ value of 6.55 × 10^−8^ M·s^−1^ at a nanocatalyst concentration of 8.28 mg·L^−1^. Of the other four synthesized types of AuNCs, the best catalytic performance for oxidizing TMB was shown for the His@AuNCs/RGO variant. It was assumed that His@AuNCs react via enzyme-like catalytic centers, when substrates and active sites of His@AuNCs are located on the RGO sheets. The “co-interaction” between the His@AuNCs and RGO increases the speed of electron transfer from TMB to oxygen, leading to enhanced catalytic activity [41].

In another example, the catalytic steady-state kinetics for CoFe_2_O_4_ CF300 was estimated using TMB and H_2_O_2_ as substrates [71]. It was shown that the PO-like reaction followed a Michaelis-Menten kinetics toward both substrates. The K_M_^app^ for TMB of the CF300 is 0.387 mM, which is practically the same as reported for PO (0.434 mM). The K_M_ for H_2_O_2_ of the CF300 is 8.89 mM, which is two-fold higher than for PO (3.7 mM), showing that CF300 has a lower affinity for H_2_O_2_.

Although in most cases the catalytic efficiency of NZs is lower compared to natural analogues, it was reported that some NZs can compete with the enzymes. For example, the highest values for any NZ reported to date belong to MnFe_2_O_4_ with a nanooctahedron morphology (see Introduction) [14,15].

Three-dimensional nanomaterials, containing confined single atoms, possess increased catalytic activity due to mutual effects of unique geometric and electronic structures of the matrix and intrinsic catalytic activity of confined atoms [65]. Shackery and coworkers [72] recently prepared a glucose oxidase-like catalyst based on cobalt hydroxide [Co(OH)_2_] nanorods on a 3D-graphene network using the chemical deposition method. The obtained nanocomposite was used for glucose detection. It was demonstrated earlier that the redox reaction of Co(OH)_2_ at the graphene surface is a diffusion-controlled electrochemical process [73]. Oxidation of Co(OH)_2_ to CoOOH is reflected by the peak at ∼0.445 V (vs. Ag/AgCl), and the reverse process corresponds to the cathodic peak at around ∼−0.08 V (vs. Ag/AgCl). A possible electrochemical reaction is as follows:

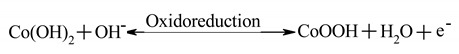


The constructed amperometric sensor demonstrated a high sensitivity for glucose (36,900 A·M^−1^·m^−2^) and a very low LOD (16 nM).

**Figure 3:**
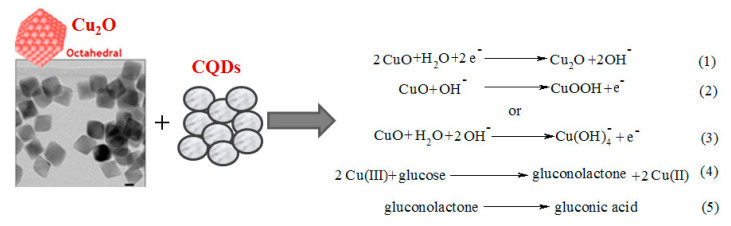
Carbon quantum dots/octahedral Cu_2_O nanocomposites for non-enzymatic glucose assay.

In another example, it was shown that octahedral cuprous oxide (Cu_2_O) in combination with carbon quantum dots (CQDs) could be an efficient electrocatalyst for the detection of glucose [74], The CQDs/octahedral Cu_2_O showed higher electrocatalysis for glucose oxidation and H_2_O_2_ reduction than the octahedral Cu_2_O. The cyclic voltammograms showed an oxidation peak at +0.6 V reflecting the conversion of Cu(II) to Cu(III) and showing high electrocatalytic ability for oxidation of glucose by the CQDs/octahedral Cu_2_O [75]. Equations (1)–(5) of the electrocatalytic oxidation reaction are presented in Figure 3.

Pirmohamed et al. [76] showed that nanoceria (CeNPs) is a redox active catalyst which possesses two oxidation states (Ce^+3^/Ce^+4^) and contains transportable lattice oxygen located on its surface which facilitates the interchangeable conversion of Ce^+4^ and Ce^+3^. Hayat et al. [77] found that the dual oxidation state and high mobility of surface oxygen are responsible for the oxidase-like activity of CeNPs toward phenolic compounds. It was shown that CeNPs exhibits the lowest K_M_^app^ value: 0.25 μM for dopamine and 180 µM for catechol, among the other nanomaterials (Table 10). For comparison, the K_M_^app^ values of the optimized enzymatic reaction using tyrosinase under the same reaction conditions were 0.3 µM and 200 µM for dopamine and catechol, respectively.

Another quantitative characteristic that evaluates the effectiveness of NZs is the half maximal inhibitory concentration (IC_50_), reflecting the ability of a substance to inhibit certain biological or biochemical functions. A nanocomposite based on gold NPs and the copper(II) complex of cysteine (GNPs/Cu-Cys) was synthesized by Dashtestani and colleagues [48]. The SOD mimetic activity of a Cu-Cys and GNPs/Cu-Cys nanocomposite was determined using the pyrogallol autoxidation inhibition assay. The half-maximal inhibitory concentration of the nanocomposite was 0.3 μg·mL^−1^, which is 3-fold higher than that of the native enzyme. However, the authors did not indicate how the concentrations of both catalysts were normalized. The IC_50_ values of the compounds indicate that all individual components related to the GNPs/Cu-Cys nanocomposite exhibited SOD mimic activity.

The enzyme-like activity can be controlled by the shape, size, crystallinity and final morphology of the NZs [78,79,80]. Liu et al. [79] showed that different nanocrystalline shapes had different PO-like activities. The activities of the NZs in descending order according to shape, can be presented in the following order: cluster spheres > triangular plates > octahedral. The activity depends on the exposure of catalytically active iron atoms or crystal planes. Two kinds of nanomaterials for O_2_^•−^ sensing were prepared: hollow carbon spheres and hollow carbon cubic nanomaterials with a size of 100 to 200 nm, respectively [47]. The electrochemical sensor prepared using the hollow carbon sphere NZ exhibited an extremely low detection limit of 1.25 nM and was successfully employed in the dynamic monitoring of O_2_^•−^ released from HeLa cells (Table 3). In another example, a detailed study for investigating the effects of particle size and morphology on the PO-like catalytic activity of magnetic cobalt ferrite (CoFe_2_O_4_) was performed [81]. CoFe_2_O_4_ NZs having different shapes (near corner-grown cubic, near cubic, polyhedron and star-like) were synthesized when varying the amounts of iron and cobalt acetylacetonates precursors and changing the reaction temperature. To increase the suspensibility of NPs in water solution, the obtained CoFe_2_O_4_ were modified with PEG-3,4-dihydroxybenzylamine. The catalytic activity was structure dependent (in descending order according to a shape): spherical > near corner-grown cubic) > star like > near cubic (> nanopolyhedrons. The kinetic studies of the obtained CoFe_2_O_4_ showed that star-like shaped CoFe_2_O_4_NPs with 4 nm-sized had the highest affinity for TMB and H_2_O_2_ compared to the other NPs obtained in the study [81].

The affinity of the NZs to substrates can be changed by a procedure of surface functionalization [82]. Ling et al. recently synthesized a new artificial nanocatalyst based on metalloporphyrinic metal organic frameworks (PMOF(Fe)) and PtNPs [83]. It was shown that Pt@PMOF(Fe)NPs showed high activity toward the oxidation of o-phenylenediamine (OPD) as a chromogenic substrate and H_2_O_2_ as the oxidant. This mechanism can be summarized in the following reaction:


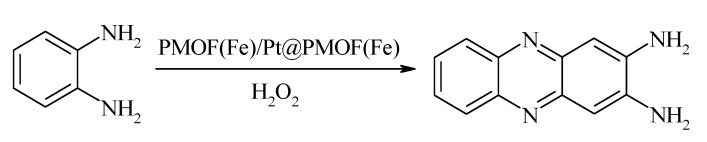


It was found that when Pt@PMOF(Fe) was mixed with H_2_O_2_ and OPD, the Fe center provided an electron to PtNPs forming high-valence Fe, due to the synergistic effects between PMOF and PtNPs, and the compound, which included Fe(IV) = O and a porphyrin π cation radical, generated via the reaction between Fe(III) and H_2_O_2_. OPD then was easily oxidized. Thus, PtNPs enhanced the PO-like activity of Pt@PMOF(Fe) [40].

The mass ratio (Ni-MOFs to Fe-MOFs) is one of the crucial factors influencing the catalytic activity of nanocatalysts. The catalytic performance of Fe-Ni-*n* (where *n* is the mass ratios) was explored by optimizing the mass ratios (Ni-MOFs@Fe-MOFs). Due to differences in the morphology and specific surface area of Ni-MOFs and Fe-MOFs, the optimal load of Ni-MOF nanosheets on octahedral Fe-MOFs was realized with the increase in *n*. When *n* = 2, the nanosheets and octahedron achieve relatively sufficient mixing. However, the *n* value is too small for the nanosheets to adequately cover every surface of the octahedrons. On the other hand, if the n is too large, there would be extra nanosheets that are not coated on the octahedrons [67].

It was shown that the activity of nanocatalysts is strongly dependent on their composition. PtRuNP alloy with a suitable degree of alloying (e.g., Pt_90_Ru_10_) can mimic oxidase (ferroxidase), PO, SOD and catalase. These enzyme-like activities correspond to the redox enzymes: oxidase and PO which catalyze oxygen and H_2_O_2_ reduction, respectively, and SOD and catalase which are responsible for disproportionation of superoxide and H_2_O_2_ decomposition, respectively. It was found that varying the composition of PtRu alloys affects their ability to facilitate electron transfer, since these reactions involve a transfer of electrons. Such dependence on the alloy composition was observed for all four enzyme-like reactions [66].

A fast, effective and low-cost method for production of ZnFe_2_O_4_ nanoparticle-decorated ZnO nanofibers using co-electrospinning and the sequence annealing process has recently been proposed [83]. It was shown that the presence of ZnFe_2_O_4_ NPs on the surface of ZnO nanofibers and formation of heterostructures significantly improved the PO-like activity compared with ZnFe_2_O_4_ NPs. Furthermore, the smaller-sized NPs had a higher activity. The obtained results suggest that the surface-to-volume ratio, the composition and the size of the NZs play a critical role in their catalytic activities [84].

## 4. Nanozymes as Peroxidase Mimetics

Peroxidases (PO, E.C. 1.11.1.7) are oxidoreductases from a variety of sources, including plants, animals and microbes, that contain an iron-porphyrin derivative (heme) in their active site and catalyze the oxidation of diverse organic compounds using H_2_O_2_ as the electron acceptor. Some of the most popular PO substrates with the highest chromogenic ability are 3,3′,5,5′-tetramethylbenzidine (TMB), 2,2′-azinobis[3-ethylbenzothiazoline-6-sulfonic acid] (ABTS) and *o*-phenylenediamine (OPD).

In the catalytic process, the Fe of the heme of natural PO provides H_2_O_2_ dissociation by changing the Fe(III) to the Fe(IV) valence state in an intermediate with high oxidative activity. The enzyme’s oxidative activity enables realization of the catalytic cycle. Therefore, if a nanomaterial is able to cause a similar electron transfer, it can be called a NZ with PO-like activity (“nanoperoxidase”). Due to the high reduction potential of POs, they are very promising for use as bioelectrocatalysts in biosensorics, fuel cell technology as well as environmental biotechnology. Despite the benefits of POs, their wide usage is still restricted, due to their fast inactivation in the presence of H_2_O_2_ under native physiological conditions. Their low thermal and environmental stability reduces the possibilities for their practical application. Screening the highly stable synthetic nanomaterials with PO-like activity for practical application in the different fields of modern technologies thus seems very promising.

**Figure 4:**
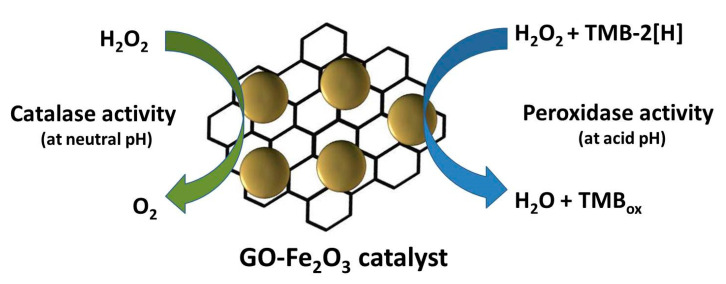
Scheme of catalytic action of graphene oxide-based Fe_2_O_3_ enzyme-like mimetic of peroxidase and catalase [121] (modified).

**Figure 5:**
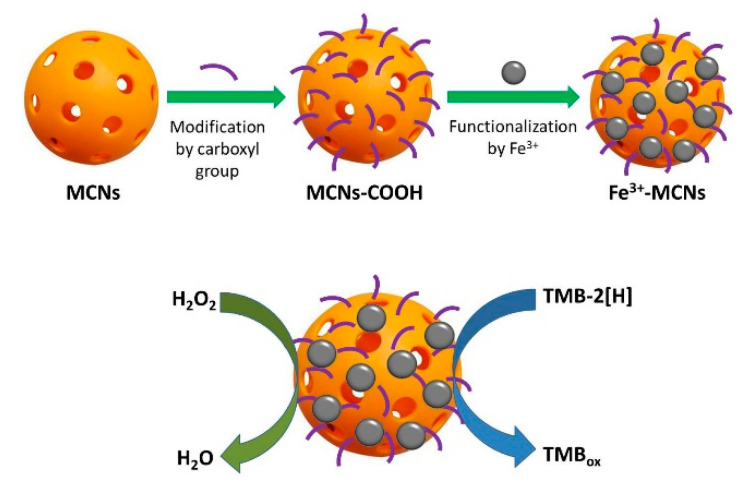
Schematic representation of the synthesis of Fe^3+^-decorated mesoporous carbon nanospheres (Fe^3+^-MCNs) and their peroxidase-mimicking catalytic activity [130] (modified).

In 2007, ferromagnetic NPs became the first reported nanomaterials with PO-like enzymatic activity [12]. Since then, the number of such materials has been growing constantly. These include different material types, such as Nafion-cytochrome *c* [85], supramolecular complexes of hydrogel [86], nanocomplexes of lanthanides [87,88,89,90,91,92], Co_3_O_4_@CeO_2_ hybrid microspheres [93], transition metal oxides and their composites [71,94,95,96,97,98,99], as well as noble metals: Au [100], Fe/Au [101], Au/Pt [102], Pt [103,104], Ru [105], Pt/Ru [60], Pd, Pd@Pt [106], Pd/Pt [107], Pd@γ-Fe_2_O_3_ [108], etc. In the last decade, special interest in bionanotechnology has focused on the use of NZs based on carbon materials such as fullerenes [109], Prussian blue [110], TiO_2_ [111] or Fe_2_O_3_/Pt-modified [112] multi-walled carbon nanotubes, hemin-graphene hybrid nanosheets [113], Pt/Ru/3D graphene foam [114], graphene oxide [115], Au/Pt/Au-graphene oxide nanosheets [116], Pd-magnetic graphene nanosheets [117], hemin-graphene-Au hybrid [118], IrO_2_ [119], Cu-Ag [120] or Fe_2_O_3_-modified graphene oxide [121] (Figure 4), Co-modified magnetic carbon [122], Co_3_O_4_ graphene composite [123], carbon nanofibers [124], graphene quantum dots [125,126,127], graphene nanotubes nickel-/nitrogen-doped and functionalyzed by PtNPs [128], carbon fiber-supported ultrathin CuAl layered double hydroxide nanosheets [129], Fe^3+^-doped mesoporous carbon nanospheres [130] (Figure 5), and many others.

The kinetic parameters of different artificial POs toward H_2_O_2_ are presented in Table 4.

H_2_O_2_ molecules are known to participate in numerous biological processes. Its analysis is routine for all newly synthesized PO mimics. Moreover, many natural enzymes (oxidases) produce H_2_O_2_ as a byproduct of their enzymatic reaction, so that detection of the target substrate can be performed bymeasuring generation of H_2_O_2_. PO-like NZs can be combined with oxidases of protein nature expanding the detection range of analytes using electrochemical biosensing. In the last decade, numerous hybrid amperometric biosensors based on PO mimetics coupled with natural enzymes, mostly oxidases (e.g., glucose oxidase, alcohol oxidase, and lactate oxidase), have been described. Because of the high pull of researches in this field, we singled out only recently described NZ-based sensors for H_2_O_2_ detection, without describing the hybrid biosensors. All analyzed PO-sensitive sensors belong to electrochemical ones with voltammetric or amperometric detection. Their main operational characteristics are presented in Table 5.

Prussian blue (PB) is one of the most effective PO mimetics. PB or iron(III) hexacyanoferrate(II) is a member of a well-documented family of synthetic coordination compounds with an extensive 300-year history. It was produced commercially in the past and used as a pigment for paints, lacquers, printing inks and laundry dyes [145,146].

PB and its analogues (PBAs) are cheap, easy to synthesize, environmentally friendly and are prospect for wide applications in different fields, including basic research and industrial purposes [10,147,148,149,150,151] as well as in medicine [24,152,153,154,155,156,157,158].

**Figure 6:**
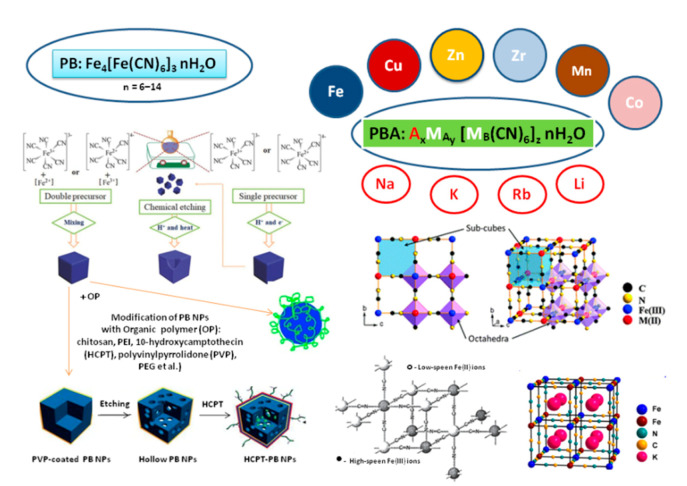
Hexacyanoferrates of transition metals: composition, structure and modification.

Despite their multifunctionality, the composition of PBAs is quite complicated and tightly depends on a method of synthesis and storage conditions. [146,149,150,151,159,160]. Insoluble PB can be described by the formula Fe_4_[Fe(CN)_6_]_3_. KFe[Fe(CN)_6_] corresponds to a colloidal solution of PB [149,151]. The general formula of hexacyanoferrate (HCF) is M_k_[Fe(CN)_6_]·xH_2_O, where M is a transition metal (Figure 6). PBAs are usually obtained via various techniques, including chemical [146,149,159,160,161,162] and alternative biological methods [163].

PBAs have gained extensive worldwide attention in the last two decades. The charge transfer through two transition metal ions in the complex-compound salt, as well as the nanosize of HCF particles, are the reasons for PBAs’ redox activity and super-magnetic properties. Due to these remarkable properties, PBAs are widely applied as NZs in biosensors [146,147,150,164,165,166,167,168] and alternative energy sources [149,160,165,168].

The enzyme-like properties of PBA in solution are difficult to analyze because of the ability of PB and PBAs to simultaneously mimic the activities of several ROS-enzymes (PO, SOD and catalase). Numerous papers (see Table 6) described the ability of PBA-NZ to react with typical ROS-enzyme substrates, such as TMB, ABTS and NADH [169].

PB and PBAs are used successfully in optical biosensors as a result of their PO-like properties [12,16,169,170,171,172,173,174,175]. The first communications concerning electrochemical reduction of H_2_O_2_ on PB-modified glassy carbon electrode were done by Itaya and colleagues [164,165]. Karyakin and colleagues published many reports over the last 25 years on the use of PB as an artificial PO in amperometric biosensors [13,150,162,176,177,178,179,180,181]. In 2000, this group named PB an “artificial PO” [177]. At the same time, a large number of other scientific groups, especially from China, worked hard on this problem [148,154,156,157,158,159,166,168,169,170,171,172,173,174].

**Figure 7:**
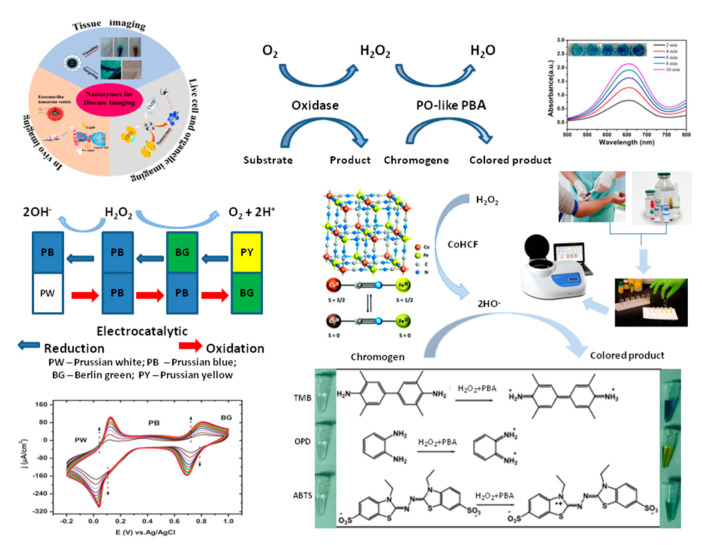
Peroxidase-like catalytic activity of Prussian blue analogues (PBAs) as a platform for the development of amperometric and colorimetric (bio)sensors.

PBAs demonstrated intrinsic PO-like activity when coupled with carbon, graphene, natural polysaccharides or synthetic polymers. This property was successfully used in electrochemical (bio)sensors (Figure 7).

Table 7 presents selected examples of PBA application as amperometric chemo-sensors on H_2_O_2_ and the analytical characteristics of the developed sensors. The main peculiarities of these catalysts are high stability, sensitivity and selectivity to H_2_O_2_ in extra-wide linear ranges. PBAs are more stable in neutral and alkaline solutions compared to PB [181], and may be used in a physiologically compatible medium containing biorecognition elements. Some PBAs show good selectivity against easily oxidizable interfering species, for example organic acids, although the electrocatalytic activity in H_2_O_2_ reduction is similar to that of PB [146,147,149].

Electrochemical biosensors have long been used as an efficient way for quantitative detection of different analytes (biomarkers) of interest. PBA-based NZs, being PO-mimetics, may comprise a promising platform for the construction of biosensors that can be applied in clinical diagnostics, theranostics, for control of therapy, cell/tissue growth and proliferation [5,6,7,8,9,10,11,146,147,148,152,170,192].

The main drawback of the many H_2_O_2_-sensitive NZ-based electrochemical sensors is the application of rather high or low working potentials. As a result, they suffer from non-selectivity. It is known that H_2_O_2_ is prone to direct auto-oxidation on electroactive surfaces (e.g., Pt) at an operational potential above +0.4 V or auto-reduction at −0.4 V or less vs. Ag/AgCl. Moreover, the real samples consisted mostly of organic compounds which are easily co-oxidized/co-reduced at the above-mentioned potentials (e.g., ascorbic or uric acids, neurotransmitters, pigments, drugs, and even glucose), consequently resulting in overestimation of the target analytes. There are only a few possibilities for decreasing this interfering impact, for example by restricting the access of the potentially interfering compounds by perm-selective membranes. The weak point of this approach is the additional diffusion limitations, resulting in deterioration of the sensor’s operational parameters. A much more successful strategy is the screening of new NZ types that work at operating potentials close to zero (0) V vs. Ag/AgCl [193].

## 5. Nanooxidases

Natural oxidases, which belong to the EC class 1.1.3, catalyze the oxidation of many substrates that contain the CH-OH group (electron donors). They use molecular oxygen as the electron acceptor, and form hydrogen peroxide as a byproduct [194]. Generally, a specific oxidase name is given according to the target oxidized substrate.

For example, glucose oxidase (GOx), alcohol oxidase (AOx), lactate oxidase (LOx), cholesterol oxidase (COx), etc. are the specific oxidases that catalyze the oxidation of glucose, ethanol, lactate, and cholesterol, respectively:

Glucose + O_2_ → Gluconic acid + H_2_O_2_ (GOx)

Alcohol + O_2_ → Aldehyde + H_2_O_2_ (AOx)

Lactate + O_2_ → Pyruvate + H_2_O_2_ (LOx)

Cholesterol + O_2_ → Cholestenone + H_2_O_2_ (COx)

Due to the generation of H_2_O_2_, natural oxidases and oxidase-like NZs can efficiently oxidize the colorless substrates (in the presence of PO) into corresponding colored products, which make them promising tools for analysis of a number of biological molecules. Many forms of nanomaterials that exhibit nonenzymatic oxidase-like catalytic activities have been reported in recent decades: ferrous metals, their oxides or bimetallic/alloys [195,196,197,198], nanocomplexes of lanthanides [199,200,201], transition metals [105,202,203], as well as noble metals and their combinations: (IrPd)/Au [204], PtNPs [205,206], Au@Pt [207], Au/Pt/Ag [208], Au-Pd NPs [209], Au/TiO_2_ [210] and many others [211]. Although the metallic GOx-like NPs yielded >99% gluconic acid, these materials suffered from high adsorption of reaction products resulting in their oxidation and inactivation [212]. The most extensively studied nanooxidases used for selective oxidation of glucose to gluconic acid are AuNPs [4,210,213,214,215,216,217,218,219,220,221,222] (see Table 8). Gold NPs are much more resistant to O_2_ compared with Pt and PdNPs and their reaction products have a lower affinity to adsorption onto the Au surface, in addition to being more active and selective under mild conditions [212]. Their main drawbacks are strong dependence of catalytic activity on a type of Au surface and nanoparticle size affected by of sintering [223]. Carbon-based materials and metal/carbon composites have also demonstrated catalytic oxidation of glucose in the presence of O_2_ [224,225,226,227] as an alternative to metallic NPs.

Unfortunately, the catalytic parameters were not determined for many of the above-mentioned nanooxidases. It is therefore not possible to compare the efficiency of GOx-mimicking nanocomposites.

**Figure 8:**
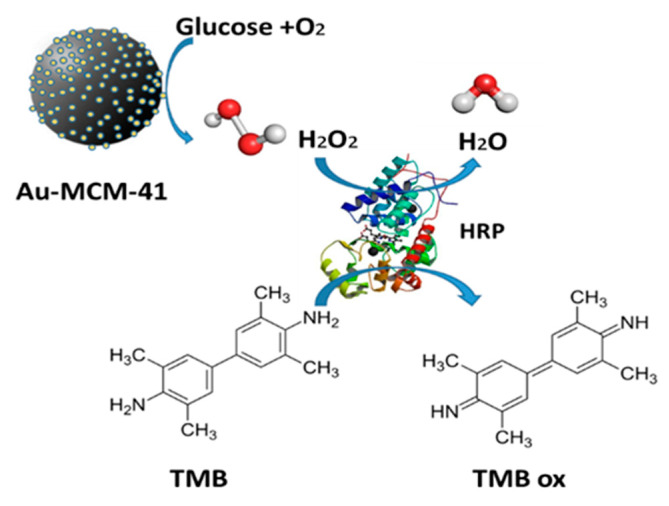
Determination of H_2_O_2_ as a product generated in the reaction of glucose oxidation [212] (modified).

Ortega-Liebana and coauthors [212] (Figure 8) described an Au-silica nanohybrid Au-MCM-41 with a determined K_M_^app^ of ~55 mM, which is significantly higher compared with the values reported for free AuNZ (K_M_^app^ ~7 mM) [213], polymer-coated gold-based mimics (K_M_ ~0.4 mM) [221], gold-supported mimicking systems (K_M_^app^ ~27 mM) [229], as well as the natural GOx (K_M_^app^ ~5 mM) [213]. Au-MCM-41 NZ showed a slightly lower affinity towards glucose as a substrate. Nevertheless, the corresponding catalytic constant k_cat_, a true catalytic parameter for comparison determined as the V_max_/concentration of catalyst ratio, is close to the value reported for natural GOx (k_cat_ ~14.2 s^−1^ vs. k_cat_ ~9.7 s^−1^, respectively) and similar to freestanding Au NPs (k_cat_ ~18.5 s^−1^) as reported by Luo and coauthors [213]. The Au-MCM-41 NZ showed a good response, possibly due to a good homogeneous distribution of active sites of Au in the mesoporous carrier, which improved their availability [212].

Some of the described NZs are characterized by double enzyme-like properties. They are named “tandem NZs” (nanomaterials with tandem enzyme-like characteristics). Ma and coauthors [222] investigated catalytic properties of single AuNPs and Ag-Au hybrid NPs as possible GOx and PO mimetics. The electrochemical experiments demonstrated that a high turnover of NZs was obtained from individual catalytic elements compared to results from ensemble-averaged measurements as a classic approach. The authors concluded that the unique increasing catalytic activity of single NZ supports is due to the high accessible surface area of monodispersed NPs and high activities of carbon-supported NP during single particle collisions on a carbon ultra-microelectrode. It was proposed as a new method for accurate characterization of NZs’ catalytic activities that opens further prospects for the design of highly efficient catalytic nanomaterials. Kou and coauthors [228] described the synthesis of β-CD@AuNPs that are characterized by simultaneous GOx-like and PO-like activities (Table 8). Han and coauthors [230] desribed the synthesis of 2D MnO_2_-based NPs with dual enzyme activities in a similar pH range. Moreover, a one-pot nonenzymatic approach was proposed for the colorimetric analysis of glucose, where the oxidation of glucose and the colorimetric detection of H_2_O_2_ are conducted simultaneously as a result of the single NZ (MnO_2_ NPs) catalysis. This method is characterised by a high sensitivity, low LOD and a short time of analysis, because of the proximity effect and in situ reaction [230]. However, the weak point of the most widely described oxidase mimics is their nonselective oxidation of a number of substrates, contrary to natural enzymes. Improving the selectivity of oxidase-like NZs is therefore a great challenge that needs to be solved before their successful application in analytical technologies.

Summarizing the above-mentioned data on nanocomposites with GOx-like activity, it is worth emphasizing that many of them (see Table 8) have a very low activity that does not enable defining them as NZs. There is an enormous difference in catalytic efficiency of the described GOx-mimetics: k_cat_ values differ more than 107-fold. Although according to BRENDA data this parameter is also very variable for natural GOs-from 0.005 to 2300 s^−1^ [231], it is obvious that improving the catalytic efficiency of synthetic NZs is a very important challenge. Nevertheless, the most effective nanooxidases (including those presented in Table 9) can be a good basis for the creation of non-enzymatic sensors for glucose analysis [72,222,223,224,225,226,227].

Many sensors for glucose detection are based on its electrochemical oxidation directly on a nanocatalyst-covered electrode have been reported (Table 9). A silicon-based amperometric non-enzymatic sensor for the glucose determination (nEGS) was described by Miao and coauthors [232]. Ni–Pd NPs adhered onto a supporter comprising the 3D ordered silicon microchannel plate (MCP) were proposed as sensing materials and were used as an electrode. The 3D structure provided ample space allowed a fast mass transport of ions/gas through the electrolyte/electrode interface, thus causing fast electrochemical reactions. The Ni–Pd/Si-MCP nanocomposite electrode showed strong electrocatalysis of glucose under alkaline conditions. The nanocomposite was characterized by a good selectivity even in the presence of high concentrations of interfering agents, excellent storage stability and reproducibility.Ye and coauthors [233] synthesized and employed heterostructured Pd-Pt core-shell nanocubic materials (NCs) as nEGSs, due to their electrocatalytic activity in glucose oxidation. These core-shell NCs with a large surface area show remarkable GOx catalytic activity and can potentially be applicable as nEGSs. Gao and coauthors [224] developed a PtNi alloy NP-graphene composite and found that the PtNi-ERGO nanocomposite-based nEGS possessed many merits in terms of high selectivity, superior resistance to poisoning, low LOD, rapid response, excellent reproducibility and stability, which outmatches the performance of any other reported Pt-based nEGSs. The nEGS retained 93.2 and 90.5% of its initial sensitivity at 10 and 50 days postpreparation, respectively. The combination of these unique characteristics has enabled the application of this new type of nanoelectrocatalyst-loaded electrodes for analysis of real human samples. Li and coauthors [225] proposed utilizing 3D porous copper foam (CF) as an electroconductive base and a precursor for a growth of CuO nanowires (NWs) in situ used for the construction of electrochemical nEGSs. CF has a high surface area due to its unique 3D porous structure, resulting in good sensitivity for glucose detection. The CuO NWs/CF-based nEGSs are characterized by good selectivity, reproducibility, repeatability and stability (Table 9).

The CuO NWs/CF based nEGSs have also been employed for glucose assay in human serum and saliva (which indicated that CuO NWs/CF are promising for noninvasive glucose detection). Li and coauthors [225] designed an electrochemical nEGS based on a novel nanostructured electrocatalyst of carbon quantum dots (CQDs)/octahedral cuprous oxide (Cu_2_O) nanocomposites [74]. Compared to octahedral Cu_2_O, the CQDs/octahedral Cu_2_O exhibited preference for electrocatalysis over glucose oxidation and H_2_O_2_ reduction. The experimental results demonstrated that nEGSs have a good potential for practical determination of glucose in real samples of biological liquids [75,236]. Ju and coauthors [226] synthesized a nanocomposite consisting of 1D CuNWs and 2D reduced graphene oxide nanosheets (CuNWs/rGO) and constructed amperometric nEGSs. Contrary to the CuNWs, the CuNWs/rGO hybrids exhibit a higher current response relative to their auto background current, indicating a stronger electrocatalytic capacity toward the oxidation of glucose. The sensor is characterized by very high sensitivity (16,250 A·M^−1^·m^−2^) and low LOD (0.2 μM). Shackery and coauthors [72] described the porous, conducting, chemically stable structure of Co(OH)_2_/3DG. The unique Co(OH)_2_ NRs electrode morphology displays a unique high sensitivity—36,900 A·M^−1^·m^−2^ with sufficient selectivity (Table 9).

The bifunctional cascade catalysis was successfully tested for the real-time colorimetric glucose detection with 0.8 μM LOD for 30 s. Alkalized graphitic carbon nitride (AKCN) exhibited perfect photoactivity for H_2_O_2_ generation at neutral pH-conditions, which are typical for natural GOx. The photocatalytic GOx-like activity of AKCN was successfully demonstrated by an in situ photoproduction of H_2_O_2_ which was proportional to the rate of glucose. The production of H_2_O_2_ exhibited a wide linear range proportional to glucose concentration (up to 0.1 M). The production of CO_2_ from the photocatalytic oxidation of glucose was negligible (2 μM), compared with that of H_2_O_2_. This indicates that the photocatalytic mineralization of glucose is inhibited at the applied conditions, and glucose is selectively phototransformed into gluconic acid on AKCN.

**Figure 9:**
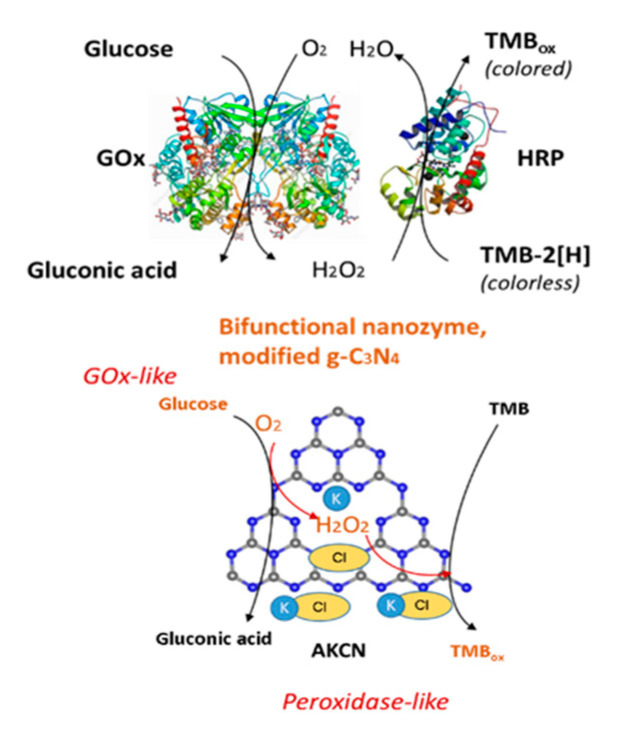
Comparison of two glucose detection systems: based on the glucose oxidase (GOx) combined with horseradish peroxidase (HRP) (above); based on synthetic NZ aerobic photocatalytic oxidation of glucose combined with in situ H_2_O_2_-production on AKCN (alkalized GCN—graphitic carbon nitride) (below) [237] (modified).

In addition to amperometric sensors, recent publications described several promising photoelectrochemical nEGSs using oxidase-like NZs. Zhang and coauthors [237] reported a synthesis of metal-free oxidase mimicking NZ based on modified graphitic carbon nitride (Figure 9). The H_2_O_2_ is generated as a result of coupled photocatalytic oxidation of glucose and O_2_ reduction under visible light irradiation with about 100% apparent quantum efficiency. The generated in situ H_2_O_2_ serves for oxidation of a chromogenic substrate on the same catalyst in a dark to complete the nonenzymatic glucose detection.

Cao and coauthors [235] recently reported the development of a photoelectrochemical glucose sensor using complicated ternary layered NPs of ITO/PbS/SiO_2_/AuNPs (ITO-indium tin oxide). Thioglycolic acid-capped PbS quantum dots that are highly sensitive to oxygen were employed as a photoelectrochemical active probe. The AuNPs were used as the GOx-like NZ for aerobic catalytic glucose oxidation. The catalysis promoted oxygen consuming, resulting in a decrease in the cathodic photocurrent. The insertion layer of SiO_2_ NPs between PbS and AuNPs could efficiently reduce the base current due to its low electroconductivity, which improved the LOD. The described sensor showed high sensitivity and good selectivity. The LR toward glucose was in the frames from 1.0 μM to 1.0 mM with 0.46 μM LOD.

Thus, glucose sensors are practically the only oxidase-like NZ-based electrochemical sensors that are currently being developed and characterized. GOx-like NZs incorporated on the electrode covered layer enhanced their catalytic power due to intrinsic catalytic activity and a synergetic effect of applied potential. In our opinion, due to additional electrocatalytic activity, such NZs can be a promising alternative for natural enzymes in the construction of electrochemical sensors. They are cost-effective, possess high sensitivity, favorable stability, reproducibility, simplicity in development and avoid complex enzymatic immobilization techniques. Unfortunately, performing a multielectron oxidation reaction in the presence of easily oxidized interfering agents has some severe constraints. For example, platinum electrodes lose their activity quickly in glucose solutions through accumulation of chemisorbed intermediates which block the electrocatalyst’s surface [238].

## 6. Laccase-Mimicking Nanozymes

**Figure 10:**
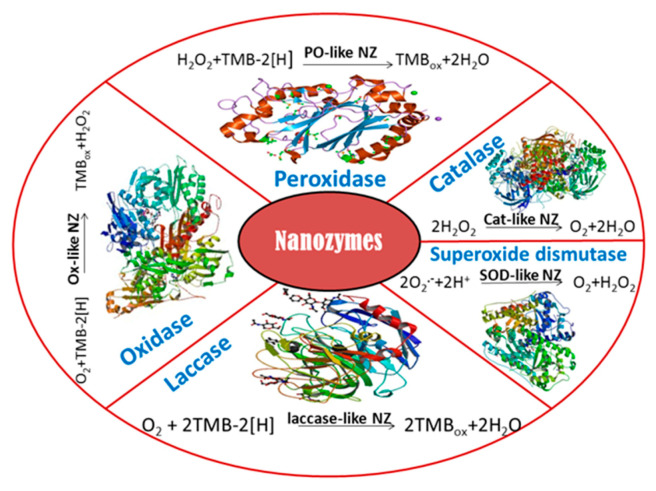
Scheme of reactions catalyzed by oxido-reductases-mimicking nanozymes (NZs).

Natural laccases [239,240,241,242] are members of the multi-copper oxidases which catalyze the single-electron oxidation of a wide range of organic substrates, such as polyamines, aryl diamines, ortho- and para-diphenols as well as polyphenols, with the subsequent four-electron reduction of molecular oxygen to water (Figure 10). Due to their activities, laccases can be used as “green” catalysts in water treatment and soil bioremediation [243,244]. However, the poor stability of natural laccases in complex environments, the difficulty of their recycling, and the high cost of the purified enzyme preparations severely hamper practical applications of this enzyme [12,245,246,247,248].

**Figure 11:**
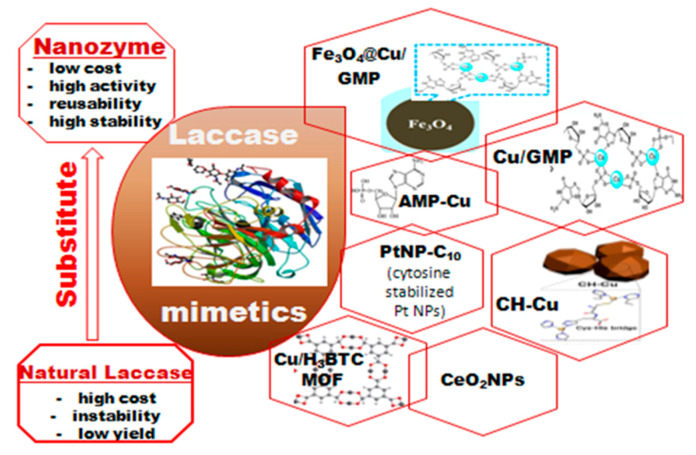
Laccase-mimicking nanozyme for oxidizing phenolic compounds [263,264,265].

Many synthetic methods for obtaining various types of laccase-like nanomaterials have been described, when most of them are based on the use of copper ions as a catalyst, because the active centers of natural laccases also contain these ions. A large number of copper-based complexes with different types of organic ligands are reported as laccase mimetics [249,250,251,252,253]. Ren and other authors [254,255,256,257] reported one-pot synthesis of copper-containing carbon dots as laccase mimics. Shams and coauthors [258] described the synthesis of Cu/H_3_BTC MOF (copper ions with 1,3,5-benzene tricarboxylic acid, H_3_BTC and metal–organic framework, MOF) possessing laccase-like activity with regard to oxidation of phenolic compounds. Cu/H_3_BTC MOF was used for quantitative detection of epinephrine. This NZ showed excellent stability under different conditions compared with natural laccase [258] (Figure 11).

Water-soluble nucleotides have a significant potential for use as ligands for different nanostructures. Nucleotide coordinated Cu^2+^ complexes were demonstrated as having laccase-like activity [259,260]. Such coordination complexes were immobilized onto magnetic NPs forming Fe_3_O_4_@Cu/nucleotide NPs [260,261]. The guanosine monophosphate (GMP) based laccase mimicking Cu/GMP NZ [262] and Fe_3_O_4_@Cu/GMP NZ [263] demonstrated excellent laccase-like catalytic activity toward high spectra of phenolic substrates, e.g., hydroquinone, naphthol, catechol, epinephrine and o-phenylenediamine. The K_M_^app^ of Cu/GMP toward 2,4-dichlorophenol was quite similar to that of natural laccase (0.59 mM vs. 0.65 mM, respectively). Although it was reported that the V_max_ of Cu/GMP was 5.4-fold higher compared with the natural enzyme, the value of the intrinsic catalytic parameter (k_cat_) was not indicated. Cu/GMP also showed better stability over pH 3–9, temperatures of 30–90 °C, and a high ionic strength of 500 mM NaCl, as well as long-term storage for 9 days. Analysis of epinephrine with Cu/GMP was nearly 16-fold more sensitive and 2400-fold more cost-effective than using natural laccase [262]. The magnetic Fe_3_O_4_@Cu/GMP NZ is able to oxidize toxic o-phenylenediamine and showed higher activity and stability compared with natural laccase [263]. However, the K_M_^app^ of laccase was 18-fold lower than that of the Fe_3_O_4_@Cu/GMP NZ, which means that laccase had a better affinity toward the substrate. On the other hand, the Vmax of Fe_3_O_4_@Cu/GMP was almost 4.2-fold higher than that of laccase (see our remark on the irrelevance of such a comparison). Fe_3_O_4_@Cu/GMP retained about 90% of its residual activity at 90 °C, with little change at pH 3–9, and showed excellent storage stability.

Huang and coauthors [264] described a laccase-mimic NZ based on copper ions and adenosine monophosphate (AMP-CuNZ) with a 15-fold higher catalytic activity than that of natural laccase (at the same mass concentration; normalization to the molar concentration of both catalysts for calculating k_cat_ was not reported). It also has a higher V_max_ and a lower K_M_^app^. The V_max_ of AMP-Cu was 4.5-fold higher than that of natural laccase (1.30 μM·min^−1^ vs. 0.28 μM·min^−1^ at the same mass concentration of both catalysts, 0.1 mg·mL^−1^), with a 4-fold lower K_M_^app^ (0.09 mM vs. 0.36 mM). The lower K_M_^app^ of AMP-Cu indicates that the simulated enzyme had a stronger affinity toward the substrate. The concentration linear range (LR) of phenolic compounds detected by AMP-CuNZ was 0.1–100 µM, and the LOD was 0.033 µM (lower than that of laccase). The AMP-Cu had good stability (over 9 days of storage) under conditions of 30–90 °C and pH > 6. AMP-Cu NZ can be used to detect a variety of phenolic compounds: phenol, hydroquinone, p-chlorophenol, resorcinol, phloroglucinol and catechol [264]. It could be predicted that due to its favorable properties, AMP-Cu NZ has a great potential for applications and can replace native laccase in biosensors.

Wang and coauthors [265] presented a new class of laccase-like NZs (denoted as CH-Cu). The electron transfer in this system was providedvia the coordination of Cu^+^/Cu^2+^ with a cysteine (Cys)-histidine (His) dipeptide. The CH-Cu has similar K_M_^app^ values to the natural enzyme (K_M_^app^ 0.42 mM and 0.41 mM, respectively) and increased V_max_ values compared to the natural enzyme (7.32 μM·min^−1^ and 6.41 μM·min^−1^, respectively, at a catalyst concentration of 0.1 mg·mL^−1^ for CH-Cu or laccase). The k_cat_ value of CH-Cu is much higher than that of laccase (1.91 × 10^4^ min^−1^ and 4.13 min^−1^, respectively), indicating a higher turnover number and catalytic efficiency of a single CH-Cu NZ particle, which has a larger number of active sites than the natural protein molecule. A similar result was found for Fe_3_O_4_ NPs with PO-mimicking activity (k_cat_ = 3.1 × 10^5^ s^−1^) [17].

The catalytic activity of NPs is dependent on the copper ions content. The authors therefore measured the catalytic activity of laccase and CH-Cu NZ with the same amount of Cu atoms. The amount of copper in laccase is 0.32 wt. % and in CH-Cu it is 38.3 wt. %. As a result, the weight ratio of laccase to NZ was 120:1 for the activity test. Due to increasing the number of active sites in a single particle, CH-Cu shows good laccase-like catalytic activity. The higher efficiency of CH-Cu compared with laccase in the degradation of chlorophenols and bisphenols was also demonstrated in a batch mode. Moreover, using CH-Cu a new method for the quantitative detection of epinephrine was developed [265].

Nanomaterials that do not contain copper could also possess laccase mimicking activity. Many metal-based catalysts have been described recently, including noble metal NPs: gold [266], platinum [267], or oxides such as iron oxide [12,17], cerium(IV) oxide [268,269], manganese oxide [270], etc.

NZs placed on Pt in combination with oligonucleotides as stabilizing agents (adenine-A10, thymine-T10, cytosine-C_10_, and guanine-G_10_) display excellent catalytic performance in oxidation of multiple substrates, including 2,4-dichlorophenol (2,4-DCP), dopamine, p-phenylenediamine, catechol and hydroquinone [267]. This kind of Pt NZs has high stability in the range of 20–90 °C and pH 3–9, which exceeds the range of native laccase. It was shown that the laccase-like activities of PtNPs are strongly associated with particle size (2.5–5 nm). PtNPs modified with the oligonucleotides cytosine (C_10_), with a particle size of 4.6 nm, showed the highest activity in the oxidation of 2,4-DCP. These NPs exhibit an apparent K_M_^app^ value of 0.12 mM toward 2,4-DCP, whereas laccase exhibits a K_M_^app^ of 0.40 mM. Enzyme-like kinetics was observed in the oxidation of 2,4-DCP catalyzed by DNA-stabilized PtNPs which have a three-fold better substrate affinity compared to natural laccase [267]. Nanozymes PtNP-C_10_ and AMP-Cu possess lower K_M_ values (0.12 mM and 0.09 mM, respectively), that indicate their better affinity toward 2,4-DCP compared to natural laccases: from *Pycnoporus sanguineus* sp. CS43 (0.224 mM) and *Trametes versicolor* (0.40 mM).

The main catalytic characteristics of the synthetic laccase mimetics and natural enzyme toward different substrates are presented in Table 10.

**Figure 12:**
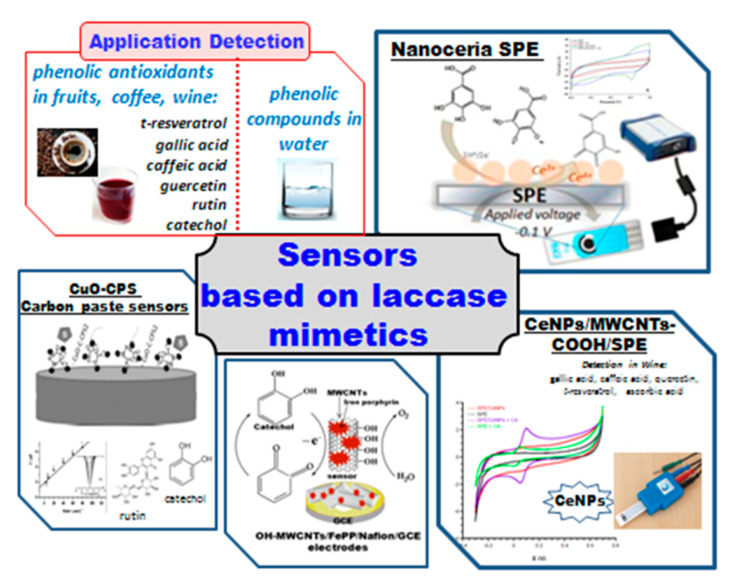
Sensors based on laccase-mimicking nanozymes of different chemical nature in detection of phenolic compounds [268,269,272,273].

Since the synthetic laccase mimetics have preferential properties compared with natural enzymes, they appear to be very promising for the development of new non-enzymatic amperometric sensors for assaying phenolic compounds (Figure 12).

Garcia and coauthors [272] reported the construction of a CPS2 (copper oxide-based carbon paste) biomimetic sensor for phenol, rutin and catechol determination in natural samples, such as dried extracts of red fruits and coffee. The enhanced sensitivity towards catechol as a model substrate correlated to the amount of incorporated copper oxide, resulting in improvement of electroactive surface area and electrocatalytic ability os the sensor. The sensor can be characterized by high sensitivity and selectivity for rutin (LR of 1 to 120 µM and LOD of 0.4 µM) and catechol (LR of 10 to 600 µM) (Table 11) that is better compared to laccase-based biosensors.

Andrei and coauthors [268] reported the construction and characterization of a disposable single-use electrochemical sensor (SPE) based on CeNPs for the detection of phenolic antioxidants. The nanoceria supported oxidation of phenolic compounds to their corresponding quinones that can be detected at the SPE surface. For analysis of reactivity of gallic acid, ascorbic acid, quercetin, caffeic acid and epicatechin the amperometric detection was performed at the working potential −0.1 V vs. Ag/AgCl. The LR for gallic acid was between 50–200 µM, with a LOD of 15.3 µM (Table 11). The electrode did not respond to glucose, tartaric acid, ethanol, citric acid and sulfur dioxide at the applied conditions. The developed CeNPs SPE electrodes are low-cost, stable and disposable, making them promising for rapid field detection of antioxidant-rich samples and for screening purposes.

Another sensor has been developed for the analysis of antioxidants in wines based on CeNPs as a biomimetic [269]. The proposed single-use electrochemical screen-printed electrode, modified by CeNPs, was applied for detection of a number of antioxidant compounds which are present in wines (Table 11). The LR of the sensor is similar, and in some cases better, than for other analogs. The sensor does not require any specific storage conditions (e.g., buffer and low temperature) as opposed to enzymatic biosensors. Furthermore, the CeNP-modified electrode has a broader LR for gallic acid and ascorbic acid [269].

Wang and coworkers [270] proposed different crystalline nanostructured manganese oxide (MnO_2_) and Mn_3_O_4_ on an electrode surface, which showed laccase-like catalytic activity toward ABTS and 17β-estradiol (E2). The best catalytic performance of the six different crystal structures for oxidizing ABTS and E2 was shown for the γ-MnO_2_ variant. ABTS oxidation by γ-MnO_2_ NPs under different pH values proved high oxidation activity of γ-MnO_2_ at pH values of 3–4. The findings add to the understanding of the laccase-like catalysts based on MnOx [270].

Bin and coauthors [273] developed a novel highly stable sensor for cost-effective and efficient catechol detection based on the functionalized surface of multi-walled carbon nanotubes (MWCNTs) and iron porphyrins (FePP). Under optimal condition, the LOD for this NZ is slightly higher than that of the laccase sensor with the broader LR (Table 11). The reproducibility test showed that OH-MWCNTs/FePP/Nafion/GCE has better reproducibility than other laccase sensors. Storage analysis demonstrated that the oxidation current maintained 98.3% of its initial output after storage at −4 °C for 35 days.

## 7. Conclusions and Potential Applications

Only a small part of the known NZs and electronanocatalysts used in the construction of electrochemical sensors is presented in this review. At present, nanozymology is progressing faster than could be described directly as a real-time review. However, we believe that the presented data will be valuable for demonstrating the preferability of a combination of novel progressive nanotechnologies with electroanalytical approaches compared to classic enzymological methods.

Enzyme-based sensors are already successful commercial products in different application fields. Using cost-effective synthetic enzyme-like nanomaterials seems to be a very promising way for sensor development. It should be noted that NZs have several limitations for their application that need to be solved. The main disadvantage of most known NZs is the lack of substrate specificity. This drawback raises concerns regarding “nanozyme” term since in some cases these nanomaterials operate like chemical or electrochemical catalysts. The second drawback is the fouling of the NZ surface due to aggressive impact or adsorption of some compounds of the real environmental or biological samples. The third is a rather low reaction rate as a result of pH-sensitivity. Many common NZs work best in an acidic pH, while the pH of biological samples is often neutral. The fourth is a limited reaction type of the current NZs that are mainly capable of mimicking oxidoreductases (oxidases, peroxidase, catalase, SOD). Known NZs are thus currently able to mimic only a small fraction of enzymes. These critical problems must be solved in order to create highly selective, stable, reliable NZs that are suitable for practical use. However, considering the high scientific interest, progress in this field will focus on the creation of novel materials with new improved catalytic as well as physic-chemical properties, maximally fitted for (bio)chemosensing devices.

## Figures and Tables

**Table 1:** Natural enzymes—biocatalysts of protein origin (except for ribozymes).

Advantages	Drawbacks
An extremely high rate of enzymatic reactions: spontaneous reactions can run for millions of years, while enzymatic ones run for milliseconds. Examples of particularly active enzymes: -Catalase (2H_2_O_2_ → 2H_2_O + O_2_) 1 molecule of the enzyme catalyzes the decay of 5 mln S per 1 min; -Carbanhydrase (CO_2_ + H_2_O ⇔ H_2_CO_3_ ⇔ H^+^ + HCO_3_^−^): 36 mln turnovers per 1 min.	Physicochemical instability to action of environmental (chemical and physical) factors. Biological instability (susceptibility to degradation by proteases).
High selectivity	High costs of isolation and purification.

**Table 2:** Advantages of nanozymes.

(1)	Availability and low preparation costs.
(2)	Physicochemical and biological stability.
(3)	High surface area.
(4)	Self-assembling activity.
(5)	Size/composition-dependent activity. Broad possibility for modification and regulation of activity.
(6)	Compatibility with biological elements.

**Table 3:** Preparation and properties of nanocatalysts and corresponding electrochemical biosensors.

Mimetic Enzyme	Enzyme-Like Activity	Preparation Method	Pore Diameter, nm	Shape	Particle Size, nm	BET, m^2^ ·g^−1^	Potential, V	Detection	Linear Range, µM	LOD, µM	References
Au/Co@HNCF	Urease	Thermal	1.2	Dodeca hedron	300–400	7.88	+0.3	Uric acid	0.1–2500	0.023	[10]
Co_0.5_Ni_0.5_Fe_2_O_4_	PO	Solvothermal		Sphere	70–130		+0.5	H_2_O_2_	0.01–1000	0.001	[25]
SOD/mesoporous SiO_2_-(L)-lysine	SOD	Hydrolyte polycondensation	3	disk	40–70	570	0.05		3.11–177	800	[27]
SOD/PMMA/PANI-Au	SOD	Electrospinning/polymerization		fiber	400–500		0.3		0.5–2.4	300	[28]
CuO-NZs	GOx PO	Thermal	2.5	Sphere	~45	20.16	+0.55	Glucose Cholesterol	5–600 1–15	0.59 0.43	[28]
GOx/PtNP/PAni/Pt		Sol-gel method		Sphere/fiber	2/100		+0.56	Glucose	10–8000	0.7	[43]
HCC/SPCE PCC/SPCE	SOD	Solid statereaction Thermal		Hollow cubic hexahedral	100–150	356 756	−0.5	Superoxide-anion O_2_^−^	0–192 192–912 0–112 112–1152	0.207 0.140	[30]
Graphene paper/AuNPs	GOx	Laser Thermal		Sphere Plate	10–150 200–400		+0.17 +0.5 +0.19 +0.4	Glucose Fructose Glucose Fructose	20–8000 40–4000 15–8000 5–4000	2.5 ≥20 2.5	[32]
	Ox SOD CAT, PO Ox							Glucose	10–1·10^4^		[37]
PCC/SPCE		Thermal		Sphere	20 ± 3		+0.4	*P. aeruginosa*	60.0–6.0 × 10^7^ CFU/mL	60.0 CFU/mL	[38]
										
AgNP/NCF/GCE		Thermal/Electrodeposition		sphere/fiber	15~20/5~8 μm		−0.5		6.96·10^−11^–72	1.66·10^−4^	[38]
			~2	Sphere	8.4	2.3	0.04–1.0	Oxygen reduction			[39]
Pt@PMOF(Fe)		Chemical reduction/Electrodeposition		Ellipsoid	300		−0.45	Glucose	100–10,000	6	[40]
His@AuNCs/RGO-GCE		Chemical reduction		Sheet	1.72		+0.8	Nitrite	1.0–7000	0.5	[41]
GOx/PtNP/PAni/Pt		Sol-gel method		Sphere/fiber	2/100		+0.56	Glucose	10–8000	0.7	[43]
Mn-MPSA-HCC	GOx	Co-precipitation		Hollow cubic	100–200		+0.75				[47]
Mn-MPSA-HCS				Hollow sphere					0–1260	0.001	
AuNPs/Cu-Cys		Chemical reduction/Co-precipitation		sphere	77		0.25		3.1–326	2.8	[48]
Fe_3_O_4_/Au@Pt-HRP-DNAzyme-Tro6	PO	Co-precipitation		Sphere	158		−0.25		4.2·10^−13^–4.2·10^−9^	3.1·10^−15^	[49]
GCE/MWCNTs-Av/RuNPs/biot-GOx	PO	Drop-coating/Electrodeposition		Tubes/sphere	1000		−0.05	Glucose	20–1230	3.3	[59]
nPtRu/AO		Electrodeposition		Sphere	5–150		−0.1	Ethanol	25–200	2.5	[60]
nPtRu/AMO								Methylamine	20–600	3	

**Table 4:** Comparison of the kinetic parameters of different types of artificial peroxidases and natural enzyme toward H_2_O_2_ in solution.

Catalyst	Concentration	K_M_^app^, mM	V_max_, μM·s^−1^	k_cat,_ s^−1^	Reference
Fe_3_O_4_ NPs	11.4 × 10^−13^ M	154.0	0.098	8.58 × 10^4^	[12]
HRP	2.5 × 10^−11^ M	3.7	0.087	3.48 × 10^3^	[12]
CoFe_2_O_4_	20 μg·mL^−1^	8.89	0.019		[71]
CeO_2_-MMT	300 μg·mL^−1^	3.4	0.010		[88]
CeO_2_ NPs	300 μg·mL^−1^	3.18	0.009		[88]
H/WS2-NSs	3.2 μg·mL^−1^	0.926	0.028		[89]
CeO_2_ NPs	40 μg·mL^−1^	0.28	0.009		[90]
BNNS@CuS	30 μg·mL^−1^	25	0.125		[92]
Co_3_O_4_@CeO_2_	50 μg·mL^−1^	7.09	0.430		[93]
Fe_3_O_4_@Cu@Cu_2_O	50 μg·mL^−1^	2.3	0.119		[95]
H_2_TCPP-γ-Fe_2_O_3_	18.5 μg·mL^−1^	21.1	1.3 × 10^−3^		[96]
γ-Fe_2_O_3_ NPs	100 μg·mL^−1^	157.2	0.013		[97]
Fe_3_O_4_@C YSNs	20 μg·mL^−1^	0.035	0.033		[99]
CuO	100 μg·mL^−1^	440	0.161		[98]
Zn-CuO	100 μg·mL^−1^	71	0.003		[98]
H_2_TCPP-CeO_2_	40 μg·mL^−1^	0.25	0.013		[90]
Pt/CeO_2_ NPs	10 μg·mL^−1^	0.21	0.085		[91]
Pt NCs	1 × 10^−4^ M	3.07	0.182	1.8 × 10^−5^	[104]
Ru NPs	10 μg·mL^−1^	2.2	0.580		[105]
Pd NPs	5.06 × 10^−12^ M	4.4	0.065	1.3 × 10^4^	[106]
Pd@Pt NPs	1.9 × 10^−12^ M	2.23	0.050	2.5 × 10^4^	[106]
Pd@γ-Fe_2_O_3_	1.35 × 10^−6^ M	0.25	0.128	9.4 × 10^−2^	[108]
C_60_[C(COOH)_2_]_2_	2 × 10^−5^ M	~50	0.003	1.6 × 10^−4^	[109]
Fe_2_O_3_/Pt/CNTs	10 μg·mL^−1^	~0.1	6 × 10^−5^		[112]
GO-COOH	40 μg·mL^−1^	3.99	0.039		[115]
H-rGO-Au	0.5 μg·mL^−1^	3.1	0.121		[118]
IrO_2_/GO	2.4 μg·mL^−1^	5.19	~0.300		[119]
Cu-Ag/rGO	5 μg·mL^−1^	8.63	0.070		[120]
GO-Fe_2_O_3_	~1.25 × 10^−1^ M	305.0	0.101	8.1 × 10^−7^	[121]
MC	10 μg·mL^−1^	0.74	0.028		[122]
CNFs	5 μg·mL^−1^	3.0	0.390	7.8 × 10^−2^ mmole·g^−1^·s^−1^	[124]
CQDs	15 μg·mL^−1^	26.77	0.306		[125]
CQDs		0.49	0.026		[126]
GQDs/CuO	70 μg·mL^−1^	0.098	0.032		[127]
CF@CuAl-LDH	50 μg·mL^−1^	0.59	0.003		[129]
Fe^3+^-MCNs	25 μg·mL^−1^	161.0	0.007		[130]

**Table 5:** The main operational characteristics of the recently described electrochemical sensors based on H_2_O_2_-sensitive nanozymes.

Catalyst/Electrode Type	Working Potential, V	Linearity, mM	LOD, μM	Sensitivity, A·M^−1^·m^−2^	Reference
Fe_3_O_4_/3D GNCs//GCE	−0.2	0.0008–0.33	0.08	2742	[131]
α-MnO2//GCE	−0.4	0.0002–0.1	0.08	5.5	[132]
AuNBP/MWCNTs//GCE	−0.5	0.005–47.3	1.50	1706	[133]
Fer/rGO-Pt//GCE	+0.1	0.0075–4.27	~0.38	3400	[134]
rGO/Pt-Ag//GCE	−0.05	0.005–1.5	0.04	6996	[135]
GBR//GCE	+0.9	0.1–10.0	48.0		[136]
CMC@Pd/Al-LDH//GCE	−0.38	0.001–0.12	0.30	163	[137]
Pt/PANI/MXene//SPCE	+0.3	0.001–7.0	1.00		[138]
CuOx/NiOy//GCE	−0.35	0.0003–9.0	0.09	2711	[139]
GDCh-NiO//RDE	+0.13	0.00001–0.0039	0.0015	1072	[140]
Pt-Pd/MoS_2_//GCE	−0.35	0.01–0.08	3.4	764	[141]
N-CNFht//GCE	−0.4	0.01–0.71	0.62	3570	[142]
Cu_2_O/PANI/rGOn//GCE	−0.2	0.0008–12.78	0.3	394	[143]
WCC//GCE	−0.4	0.05–1.0	0.006	67	[144]

**Table 6:** The main kinetic characteristics of the PBA-based PO-like nanozymes in solution.

PBA	Concentration	Chromo-Gene	K_M_^app^, mM	V_max_, μM·min^−1^	k_cat_, nmol·µg^−1^·min^−1^	LR, μM	Reference
PB/γ-Fe_2_O_3_	20 µg·mL^−1^	TMB	323.6	1.17	0.059		[12]
VOxBG hydrogel	5 µg·mL^−1^	TMB	20	0.045	0.009		[161]
PO		TMB	3.7	0.009			[161]
PB-MIL-101 (Fe)	200 µg·mL^−1^	TMB	0.058	1.32	0.0066	2.4–100	[166]
MoS xNi-Fe		TMB					[169]
PB, soluble form	6 µg·mL^−1^	TMB	14.7	0.012	0.002		[170]
PS@Au@PB	300 µg·mL^−1^	TMB	0.17	0.38	0.001		[171]
Au@HMPB (40 °C)	40 µg·mL^−1^	TMB	88.72	2.50	0.063		[172]
PB	0.2 µM	ABTS	0.028				[173]
PB-Ferritin PB/Fe_2_O_3_	0.74 nM 0.74 nM 0.31 nM	TMB ABTS TMB	11.984 0.537 323.6	43.2 0.36 70.2	58.38 × 10^3^ min^−1^ 0.49 × 10^3^ min^−1^ 226.5 × 10^3^ min^−1^		[174]
MWCNTs-PB			1.33	6.6	0	1–1500	[175]

**Table 7:** Amperometric H_2_O_2_-sensitive sensors based on PBA as a PO-like nanozyme.

Electrode	Working Potential, V	Nanozyme	Sensitivity, A·M^−1^·m^−2^	LOD, μM	Linear Range, μM	Reference
GCE	−0.05	PB/BG AuNPs-PB/BG	2850 11243		4–83,000 9.2–8100	[167]
GCE	0.65	MnPBA	1472	3	3–8610	[168]
GCE	−0.3	MoS xNi-Fe PBA			0.1–2500	[169]
GCE	0.18	PB	10,000/20,000	1	1–5000	[176]
GCE	0.05	PB	6000	0.1	0.1–100	[177]
GCE/	0.0	Ni-FePBA	18,000		up to 100	[178]
DBD DBD GE	−0.05 −0.05 −0.05	PB Ni-FePBA PB/NZ	2100 1500 4500	0.5	0.5–1000	[179]
Planar screen-printed		Ni-PB	3500		0.1–1000	[180]
Carbon planar screen-printed	0.0	PB/film PB/NZ	6500 8500		up to 500 up to 500	[181]
GCE	0.0	PNAANI–PB	5073	0.07	1–1000	[182]
Graphene		CoPBA		0.007	5–1200	[183]
Graphene nanocomposite		CoPBA		0.1	0.6–380	[184]
Graphite-string	0.05		6413		30–1000	[185]
Graphite paste		Cu-FePBA Ni-FePBA	2030 1130	0.2 2	0.5–1000 2–1000	[186]
Nanoporous gold film		PB	7080	0.22	1–17,000	[187]
GCE		Ni-FePBA	0.192 A/M	1		[188]
GCE	0.33	Ni–Fe PBA-HNCs	361.3	0.291	0.1–20,000	[189]
GE		Thionine-NiPBA		0.557	1.67–1110	[190]
CNE		PB	500,000		10–3000	[191]

**Table 8:** Comparison of the kinetic parameters of different types of artificial GOx and natural enzyme toward glucose in solution.

Catalyst	Concentration	K_M_^app^, mM	V_max_, μM·s^−1^	k_cat,_ s^−1^	Reference
Au/MCM-41	0.025 mg·mL^−1^	55.2	18.0	14.2	[212]
AuNPs	34 × 10^−9^ M	6.97	0.63	18.52	[213]
GOx	34 × 10^−9^ M	5.0	0.69	9.7	[213]
AuNPs	0.05 mg·mL^−1^	0.41	0.10		[221]
AuNPs-MIP	0.05 mg·mL^−1^	0.18	0.42	3.76 × 10^−7^ mmole·g^−1^·s^−1^	[221]
AuNPs-PFOP	0.05 mg·mL^−1^	0.09	0.58	5.21 × 10^−7^ mmole·g^−1^·s^−1^	[221]
AuNP	2 × 10^−9^ M			4.5 × 10^7^	[222]
β-CD@AuNPs		9.60	1.80 × 10^−2^		[228]
					
EMSN-AuNPs	562.0 mg·mL^−1^	26.2	0.53		[229]
MnO_2_NFs	0.02 mg·mL^−1^	21	0.43		[230]
					

**Table 9:** Comparison of the main operational properties of glucose-sensitive amperometric sensors based on GOx-like nanozymes.

Electrode Material	Potential, V	Linearity, mM	LOD, μM	Sensitivity, A·M^−1^·m^−2^	Selectivity	Reference
3DG/Co(OH)_2_	+0.6	0.1–10	0.016	36900	AA, UA, fructose, lactose, urea	[72]
Au/MWCNTs	+0.15	0.01–36.0	3.0	1012	DA, UA, AA, fructose, saccharose, maltose, Ca^2+^, Cl^−^	[133]
PtNi-ERGO	−0.35	0.01–35	10	204.2	AA, UA, urea, fructose	[224]
CuO NW/CF	+0.35	0.001–18.8	0.3	22174	AA, UA, DA, lactose, sucrose, maltose	[225]
Octahedral Cu_2_O	+0.6	0.3–4.1	128	2410	AA, UA, DA, NaCl	[225]
CQDs/octahedral Cu_2_O	+0.6	0.02–4.3	8.4	2980	AA, UA, DA, NaCl	[225]
CuNWs/rGO	+0.58	0.01–11	0.2	16250	AA, UA, DA, AP, fructose, sucrose	[226]
AKCN	+0.7		0.8		fructose, lactose, maltose	[227]
β-CD@AuNPs	−0.05				Cu^2+^, Al^3+^, UA, AA, guanine, guanosine	[228]
Ni–Pd/Si-MCP	−0.1			0.081 A·M^−1^	AA	[232]
Pd-Pt core-shell NCs	−0.05	0.3–6.8	41.1	1700		[233]
Pt NPs	−0.05	0.3–5.2	91.8	457		[233]
Cu_2_O/GNs	+0.1	0.3–3.3	3.3	2850		[234]
Cu_2_O nanocubes	+0.1		5.9	2000		[234]
ITO/PbS/SiO_2_/AuNPs	−0.2	0.001–1	0.46		AA, UA, L-Cys, lactose, maltose, sucrose	[235]

**Table 10:** The main catalytic characteristics of the synthetic laccase mimetics and natural enzyme toward typical substrates.

Chemo/Biocatalyst	Substrate	K_M_^app^, mM	V_max_, mM·min^−1^	NP Concentration, mg·mL^−1^	Reference
CeO_2_NPs	Dopamine Catechol	0.25 × 10^−3^ 0.180			[77]
Cu/H_3_BTC MOF	Epinephrine	0.068	94 × 10^−3^	0.1	[258,262]
Laccase Cu/GMP Laccase	Epinephrine 2,4-Dichlorophenol	0.062 0.59 0.65	5.81 × 10^−3^ 0.83 0.15	0.1 0.1 0.1	
AMP-Cu	2,4-Dichlorophenol	0.09	1.3 × 10^−3^	0.1	[264]
Laccase	2,4-Dichlorophenol	0.36	0.29 × 10^−3^	0.1	
CH-Cu Cys-His dipeptide	Epinephrine/2,4-dichlorophenol	0.58 0.42	2.74 × 10^−2^ 7.32 × 10^−3^	0.1	[265]
Laccase	Epinephrine/2,4-Dichlorophenol	0.16 0.41	3.10 × 10^−3^ 6.41 × 10^−3^	0.1	
PtNP-C_10_ (cytosine stabilized Pt NPs)	2,4-Dichlorophenol	0.12	8.49 × 10^−3^	0.01	[267]
Laccase from *Trametes versicolor*	2,4-Dichlorophenol	0.40	3.51 × 10^−3^	0.16	
Laccase from *Pycnoporus sanguineus* sp. CS43	2,4-Dichlorophenol	0.224	2.21 × 10^−3^		[271]

**Table 11:** Analytical characteristics of the amperometric sensors based on synthetic laccase mimetics and natural enzyme toward typical substrates.

Sensor	Analyte	Linear Range, μM	LOD, μM	Reference
CeNPs	Gallic acid	2–20	1.5	[268]
	Caffeic acid	50–200	15.3	
	Quercetine	20–200	8.6	
	Ascorbic acid	0.5–20	0.4	
CeNPs/MWCNTs-COOH/SPE	Gallic acid	25–50	7	[269]
	Caffeic acid	33–100	10	
	Quercetin	25–100	8	
	*t*-Resveratrol	25–50	8	
	Ascorbic acid	25–100	7	
Laccase/Fc/SPE	Caffeic acid	2.0–30.0	1.6	[269]
Laccase/PAP/SWCNTs/SPE	Gallic acid	0.53–96		[269]
Laccase immobilized in polyzetidine prepolymer and SWCNTs	Gallic acid	0.53–96		[269]
CuO-C-CPS2	Rutin Catechol	1 to 120 10 to 600	0.4 2.0	[272]
Laccase on CP	Catechol	20 to 700	4.5	[272]
FePP modified multi-walled carbon nanotubes (OH-MWCNTs/FePP/Nafion/GCE)	Catechol	65 to 1600	3.75	[273]

## Abbreviations

AA	Ascorbic acid
ABTS	2,2’-Azinobis-(3-ethylbenzthiazoline-6-sulphonate)
AKCN	Alkalized graphitic carbon nitride
AMP	Adenosine monophosphate
AOx	Alcohol oxidase
AP	Acetamidophenol
AuNPs	Gold nanoparticles
Au/Co@HNCF	Gold/cobalt nanoporous carbon framework
AuNBP/MWCNTs Fer/rGO	Gold nanobipyramids supported by multi-walled carbon nanotubes ferumoxytol and reduced graphene oxide
BET	Brunauer-Emmett-Teller
BG	Bucky gel consisting of carbon nanotubes and ionic liquid
biot-GOx	Biotinylated glucose oxidase
BNNS@CuS	Boron nitride nanosheet and copper sulfide nanohybrids
BSA	Bovine serum albumin
C_60_[C(COOH)_2_]_2_	C_60_-carboxyfullerene
CeNPs	Nanoceria particles
CF@CuAl-LDH	Carbon fiber-supported ultrathin cual layered double hydroxides (LDH) nanosheets
CF-H-Au	Carbon microfibers-hemin-gold nanoparticles
CMC@Pd/Al-LDH	Pd/Al layered double hydroxide/carboxymethyl cellulose nanocomposite;
CNE	Carbon nanoelectrodes fabricated within a quartz nanopipette and electrochemically etched
CNFs	Carbon nanofibers
CNTs	Multi-walled carbon nanotubes
COx	Cholesterol oxidase
CP	Carbon paste
CPS	Carbon paste sensors
CQDs	Carbon quantum dots
cTnI	Cardiac troponin I
CTMB	Cetyltrimethylammonium bromide
Cu-Cys	Copper(II) complex of cysteine
Cu-MOG	Cu-based metal-organic gel
CuNWs/rGO	One-dimensional copper nanowires and two-dimensional reduced graphene oxide nanosheets
DA	Dopamine
2,4-DCP	2,4-Dichlorophenol
3D GNCs	3D graphene-supported quantum dots
3DG	Three-dimensional graphene
DBD	Diamond Boron-doped
EMSN-AuNPs	Expanded mesoporous silica-encapsulated gold nanoparticles
ENC	Electronanocatalyst
ERGO	Electrochemically reduced graphene oxide
FePP	Iron porphyrins
GBR	Metal-free brominated graphene
GCE	Glassy carbon electrode
GCE/MWCNTs-Av/RuNPs/biot-GOx	Glassy carbon electrode/avidin-functionalized multi-walled carbon/nanotubes/Ru nanoparticles/biotinylated glucose oxidase
GDCh	Glucose derived sheet-like carbons
GE	Graphite electrode
GNP/Cu-Cys	Gold nanoparticles with the copper(II) complex of cysteine
GNPs	Gold nanoparticles
GNs	Graphene nanosheets
GO	Graphene oxide
GOx	Glucose oxidase
GOx/PtNP/PAni/Pt	Glucose Sensor Based on Pt Nanoparticle/Polyaniline Hydrogel
H_2_TCPP	*meso*-Tetrakis(4-carboxyphenyl)-porphyrin
HCC	Carbon cubic nanomaterial
HCF	Hexacyanoferrate
h-CuS NCs	Hollow copper sulfide nanocubes
H-GNs	Hemin-graphene hybrid nanosheets
His@AuNCs	Histidine-capped gold nanoclusters
HNCs	Hollow nanocubes
H-rGO-Au	Hemin-graphene-gold nanoparticles
HRP	Horseradish peroxidase
H/WS2-NSs	Hemin-functionalized/Tungsten disulfide nanosheets
ITO	Indium tin oxide
L-Cys	L-Cysteine
LOD	Limit of detection
LOx	Lactate oxidase
LR	Linear range
MCM-41	Mobil composition of matter No. 41
MCNs	Mesoporous carbon nanospheres
MCP	Microchannel plate
MIP	Molecularly imprinted polymer
MMT	Montmorillonite
MC	Magnetic carbon
Mn-MPSA-HCC	Mn nanomaterials: hollow carbon cubic
Mn-MPSA-HCS	Mn nanomaterials: hollow carbon sphere
MOFs	Metal-Organic Frameworks
MWCNTs	Multi-walled carbon nanotubes
MWCNTs-Av	Avidin-functionalized multi-walled carbon nanotubes
NCs	Nanoclusters
NCF	Nitrogen doped cotton carbon fiber composite
N-CNFht	Nitrogen-doped carbon nanofibers
NFs	Nanoflakes
NPs	Nanoparticles
NTH	Nanotetrahedron
NW/CF	Nanowires/copper foam
NZ	Nanozyme
OPD	*o*-Phenylenediamine
PAni	Polyaniline
PANI/MXene	Polyaniline and Ti_3_C_2_T_x_
PB	Prussian Blue
PEG-HCCs	Poly(ethylene glycolated) hydrophilic carbon clusters
PFOP	Heptadecafluoro-*n*-octyl bromide
PMMA	Poly(methylmethacrylate)
PNAANI	Poly(N-acetylaniline)
PO	Natural horseradish peroxidase
RDE	Rotating disk electrode
RGO	Reduced graphene oxide
rGO	Reduced graphene oxide
SOD	Superoxide dismutase
SPCE	Screen-printed carbon electrode
TMB	3,5,3′,5′-Tetramethylbenzidine
UA	Uric acid
WCC	Tungsten carbide decorated by cobalt nanoparticles
YSNs	Yolk-shell nanostructures
ZIF	Zeolitic imidazolate framework
β-CD	β-Cyclodextrin
